# Identifying Transcription Factors Distinguishing Regenerating Cardiomyocytes in the Zebrafish Heart Using Single-Cell Transcriptomics

**DOI:** 10.3390/cells15161455

**Published:** 2026-08-13

**Authors:** Ditte Gry Ellman, Anny Carolline Silva Oliveira, Kristian Skriver Andersen, Eva Bang Harvald, Wolfgang Hofmeister, Sara Thornby Bak, Sabrina Bech Mathiesen, Ibrahim Mohamad Slaiman, Helene Juul Belling, Azra Smajic, Christina Dühring Fenger, Mark Burton, Mads Thomassen, Charlotte Harken Jensen, Elke Annette Ober, Ditte Caroline Andersen

**Affiliations:** 1 Andersen Group, Department of Clinical Biochemistry, Odense University Hospital, 5000 Odense, Denmark; 2 Clinical Institute, University of Southern Denmark, 5230 Odense, Denmark; 3 Faculty of Health and Medical Sciences, Novo Nordisk Foundation Center for Stem Cell Biology, 2200 København, Denmark; 4 Amplexa Genetics, 5000 Odense, Denmark; 5 Department of Clinical Genetics, Odense University Hospital, 5000 Odense, Denmark

**Keywords:** heart regeneration, zebrafish, cardiomyocyte proliferation/-cycling, single cell RNA sequencing, mouse, transcription factors

## Abstract

**Highlights:**

**What are the main findings?**

- Regenerating cardiomyocyte numbers peak 7–14 days after zebrafish heart injury.
- ScRNA-seq defines transcription factor networks in regenerating zebrafish cardiomyocytes.

**What are the implications of the main findings?**

- Insm1 or Tead1 overexpression doubles the number of cycling neonatal mouse cardiomyocytes.
- Knowledge of zebrafish heart regeneration may be translated to mammals.

**Abstract:**

Mammalian hearts exhibit limited regeneration, whereas zebrafish hearts present a remarkable capacity to regenerate upon injury through cardiomyocyte (CM) proliferation. Thus, understanding genes and especially transcription factors (TFs) enabling zebrafish CM regeneration may open new avenues for human heart repair. Herein, we injured zebrafish hearts by apex resection (AR) and found that the number of cycling CMs peaked around 7–14 days post-injury (dpi). Single-photon confocal imaging of 3D zebrafish hearts confirmed cycling cardiac cells throughout the heart at 7 dpi, while flow cytometry estimated that 1–2% of CMs were cycling. High-resolution single-cell RNA sequencing identified a CM cluster exclusively present after AR, whereas differential gene expression profiling identified genes defining this AR-specific CM cluster. Yet, overexpression of a single gene hit, Anxa2a, in mouse CMs did not override the inability of mammalian CMs to re-enter cell cycling. Instead, we outlined predicted upstream TFs responsible for the observed gene expression profile in the AR-specific CMs and, by proof-of-concept, overexpressed two predicted TF hits, Insm1 (Insulinoma-associated protein 1) and Tead1 (TEA Domain transcription factor 1) in mouse CMs. Notably, both Insm1 and Tead1 doubled the number of cycling mouse CMs, and despite that this induction was modest, CyclinB1 and Aurkb levels increased, pointing towards mouse CM division rather than a stimulation of polyploidy. In summary, we provide a new network of TFs predicted to regulate an AR-specific CM population in zebrafish that may be further explored to unravel zebrafish heart regeneration and eventually utilized for inducing CM proliferation in the mammalian heart.

## 1. Introduction

Myocardial infarction (MI) in humans and mammals, in general, results in a substantial number of dying cardiomyocytes (CMs), which are not replenished; instead, a fibrotic scar is formed [1,2]. This often leads to reduced heart function and complications contributing to an increase in morbidity and mortality. In contrast to humans and mice, zebrafish hearts present a remarkable capacity for endogenous regeneration upon injury [3] through re-entry of adult CMs into the cell cycle [3,4], which then substitutes the lost CMs. Zebrafish are, therefore, a valuable source for knowledge and understanding of the processes involved, such as CM cycle re-entry and further proliferation [3,4,5], and may uncover factors to be used for induction of CM proliferation in the human heart after MI. In the zebrafish heart, CM proliferation occurs predominantly at the injury border and seems to peak 7–14 days post-injury (dpi) [5]. Sarcomere disassembly has been suggested to occur partially in the regenerating CMs with a concomitant downregulation of structural myosin, and an upregulation of embryonic myosin [6,7]. In parallel, embryonically expressed cardiac genes are activated in these CMs, including *gata5*, *tbx5*, *nkx2.5*, *tbx20*, *hand2* [8], *gata4* [4,9] and *sox10* [10]. Indeed, heart regeneration is complex and seems to include several embryonic signaling pathways such as BMP signaling [11], Hedgehog signaling [12,13], the Nrg1/Erbb- [14], Fgf- [15], Jak1/stat3 pathways [16], Notch activation [17,18,19], TGFβ signaling [12,20], NF-κβ activation [21], and Igf- [22], Vitamin D- [23], *mps1-*, p38- [24] and *plk1-* [25] and retinoic acid signaling [26]. However, despite these comprehensive efforts, our knowledge on factors specifically responsible for CM proliferation and regeneration is still limited, which challenges the strategy of human heart repair through forced CM proliferation.

More recently, some systematic approaches have been adopted and applied to delineate factors, including transcription factors (TFs) playing important roles in zebrafish CM proliferation [11,27,28,29,30,31,32,33,34]. However, only a few studies [28,33] have exploited the power of single-cell RNA sequencing (scRNA-seq) to uncover specifically zebrafish single CM transcriptomics during adult heart regeneration. Through a cell sorting approach to distinguish between proliferating CMs and other cell types prior to RNA sequencing of 352 cells in the zebrafish cryoinjury model, Honkoop et al. [28] observed that proliferating and non-proliferating CMs possess different transcriptomic profiles, and that proliferating CMs resemble embryonic CMs [28]. Wu et al. [33] used the zebrafish line *Tg*(*vmhc*:*mCherry-NTR*; *amhc:EGFP*) and sorted atrial CMs and ventricular CMs prior to RNA sequencing of 1581 cells in genetically ablated zebrafish embryos. As such, they found Angpt4 as a key regulator of CM proliferation and regeneration [33]. While these studies contribute to solving the enigma of zebrafish CM proliferation, the number of CMs identified is relatively limited [28,33], which limits upstream analysis such as the prediction of TFs involved in dictating entire regeneration CM programs. Moreover, each zebrafish injury model seems to possess its own transcriptomic profile [35], suggesting that CM transcriptomics also may be quite different between the injury models (cryoinjury, apex resection (AR), and genetic ablation) of zebrafish heart regeneration. Until now, we have only identified scRNA-seq studies utilizing the cryoinjury approach [33,36,37] where CM proliferation is slower than in ablation and AR.

Recently, we increased scRNA-seq resolution specifically for CMs in mice around birth, which enabled us to identify TF networks involved in CM proliferation and polyploidy [38]. In the present study, we, therefore, set out to exploit and build on top of this approach to systematically evaluate TFs predicted to define regenerating zebrafish CMs after AR to identify potential new knowledge that may be translated in mammals to induce CM proliferation.

## 2. Materials and Methods

### 2.1. Animals

Adult zebrafish were kept and raised according to general guidelines using recirculating TECHNIPLAST systems (Techniplast, Buguggiate, Italy) (28 °C, 14/10 h light/dark cycle, and fed twice daily). Zebrafish lines used for resection and single-cell studies were wildtype AB or *Tg*(*cmlc2*:GFP) [39] lines. Only fish at least 3 months of age were used.

C57BL/6 mice were obtained from Taconic Europe (Ejby, Denmark) and group housed at the Biomedical Laboratory, University of Southern Denmark. For breeding, a female and a male mouse were housed together and P0 pups were obtained from different breeding pairs. All mice were kept in a 12/12-h light/dark cycle and fed ad libitum.

In the present study, sex was not registered since female and male zebrafish heart regeneration appears, with few exceptions [40], to be similar. Furthermore, the regenerative potential of CMs from male and female neonatal mice was considered to be the same.

All experiments involving animals were authorized and performed in line with the ethical guidelines approved by the Danish Animal Inspectorate under the Ministry of Food and Agriculture (J. no.: 2016-15-0201-00874 + 2022-15-0201-01119 + 2018-15-0201-01431) for zebrafish experiments, and (J. no.: 2016-15-0201-00941 and 2021-15-0201-01026) for mice experiments.

### 2.2. Apex Resections and Edu Injection

Apex resection (AR) was performed as outlined in Ellman et al. [41]. Briefly, adult zebrafish were anesthetized in Ethyl 3-aminobenzoate methansulfonate (Tricaine, MS-222, E10521, Sigma-Aldrich, Søborg, Denmark). Under stereomicroscopy, a small incision was made ventrally in the skin above the heart and the pericardial cavity was opened. The ventricle was exposed and 10–20% of the ventricle apex was cut off. For Sham surgery, a small incision was made, the pericardial cavity opened, and the ventricle was exposed as above. Any bleeding was stopped with pressure applied using a cotton bud. Zebrafish were returned to fresh 28 °C aquarium water after surgery and air bubbled over gills using a pipette. For experiments investigating 5-Ethynyl-2′-deoxyuridine (EdU)-incorporation, zebrafish were injected with EdU (Invitrogen, Thermo Fisher Scientific, Odense, Denmark) twenty-four hours prior to euthanasia. Zebrafish were anesthetized in Tricaine and placed dorsal side down on a damp sponge. Zebrafish were injected intraperitoneally with 10 μL of 10 mM EdU using a 30-gauge X ½ inch needle (Sterican, Hounisen, Skanderborg, Denmark) with a 1 mL syringe (BD Plastikpak, Mediq Danmark, Frederiksberg, Denmark). After injection, the zebrafish were placed in 28 °C system water until recovery.

### 2.3. Dissection of Hearts and Cryo-Sectioning

Zebrafish were euthanized using an ice water bath and placed on a sponge with their ventral side facing up. Under stereomicroscopy, an incision was made above the heart, and the operculum was cut open, allowing easy dissection of the heart, which was immediately placed in cold phosphate buffer saline (PBS). Dissected hearts were then placed and orientated with the front down in O.C.T. compound (TissueTek, cat. no. 4583, Sakura Finetek Europe, Alphen aan den Rijn, Netherlands) and frozen using Isopentane (cat. no. 270342, Sigma-Aldrich) on dry ice or, alternatively, treated in a sucrose gradient prior to embedding. Samples were stored at −80 °C until cryo-sectioning. Cryo-embedded hearts were sectioned using a cryostat (Leica CM1860 UV, Vedbæk, Denmark), with a section thickness of 10 μm.

### 2.4. Immunohistochemistry

Sections from EdU injected zebrafish were immuno-stained as previously described in Andersen et al. [42]. In brief, sections were fixed in 10% neutral buffered formalin (NBF, HT501128, Sigma-Aldrich), permeabilized in 0.5% Triton X-100 (Sigma-Aldrich) in Tris-buffered saline (TBS), blocked with TBS containing 2% Bovine serum albumin (BSA, VWR; TBS/2% BSA), before overnight incubation at 4 °C with primary antibody (Mouse anti-Myosin Heavy Chain 1 (MYH1), MF20-c, IgG2b,k 1:200, DSHB) diluted in TBS/1% BSA. All intermediate washes were performed in TBS. Sections were incubated with EdU Click-IT reaction Alexa 488 kit (Click-iT EdU Cell Proliferation Kit for Imaging, Alexa Fluor 488 dye, C10337, Invitrogen) according to the manufacturer’s protocol followed by incubation at room temperature with secondary antibody (Donkey anti-mouse IgG Alexa Fluor 555, 1:200, A31570, Invitrogen) diluted in TBS and mounting with DAPI Antifade mounting medium (Vectashield, Vector, H-1200, Thermo Fisher Scientific, Odense, Denmark).

### 2.5. Quantification of EdU^+^ Cardiomyocytes

For quantification of the percentages of EdU^+^ CMs 1, 4, 7, 14, 21, 30, and 60 days after Sham- or AR-surgery, single-photon confocal images were obtained (Olympus BX61WI, Søborg, Denmark) near the apex. Camera settings were kept constant for all pictures. The quantification was performed using the Imaris software (Bitplane Scientific Software; version 9.2.1), where an EdU^+^ CM was defined as a MYH1^+^ cell with an EdU^+^/DAPI^+^ nucleus. EdU^−^ CMs were also identified to establish the percentages of EdU^+^ CMs. Counting was performed on 3–4 randomly selected sections under blinded conditions and on areas ranging from 0.298 to 0.340 mm^2^ and 0.336 to 0.342 mm^2^ in sham and AR zebrafish hearts, respectively.

### 2.6. Preparation of Cardiomyocytes for Flow Cytometry/

Seven days after Sham- or AR-surgery, hearts were dissected as described above and enzymatically dissociated with the semiautomatic GentleMACS tissue dissociation system with heaters (MACS Miltenyi Biotec, Bergisch Gladbach, Germany; Neonatal Heart Dissociation Kit 130-098-373) according to the manufacturer’s instructions. Cells were washed in Hank’s Buffered Saline Solution (HBSS) containing 5% fetal bovine serum (FBS, Lonza, BioNordika, Herlev, Denmark) (HBSS/5% FBS) and fixed in 4% paraformaldehyde (PFA, Sigma-Aldrich) before being washed in HBSS/5% FBS. Cells were stained and analyzed as previously described [38]. Antibodies used were mouse anti-MYH1 (IgG2b,k 1:300, MF20-c, DSHB) and donkey anti-mouse IgG Alexa Fluor 488 (1:200, A21202, Invitrogen). For detection of EdU incorporation, an EdU Click-it reaction cocktail (Invitrogen, C10419) was used according to the manufacturer’s protocol.

### 2.7. CUBIC Tissue Clearing of Zebrafish Hearts

At the seventh day after Sham- or AR-surgery, zebrafish were sedated in 0.03% Tricaine solution, the heart was carefully dissected and placed in a small Petri dish containing PBS. Afterwards, the hearts were placed in a 2 mL Eppendorf tube with 500 μL 10% NBF and incubated for 4 h at room temperature (RT) for tissue fixation. Following incubation, the samples were washed in PBS and placed in a 2 mL Eppendorf tube with PBS/0.05% sodium azide (VWR) for long-term storage at 4 °C until starting the CUBIC tissue clearing protocol.

Tissue clearing of whole hearts was performed as previously published using the CUBIC (Clear, Unobstructed Brain/body Imaging cocktails and Computational analysis) protocol [43] with some modifications. Hearts were washed in TBS for 3× 5 min with gentle agitation and transferred to a 2 mL Eppendorf tube with Cubic Reagent-1 (Cubic-R1—25% Urea, 25% Quadrol (N,N,N′,N′-Tetrakis(2-Hydroxypropyl)ethylenediamine), and 15% Triton X-100 in ultrapure type I water, Sigma-Aldrich) diluted 1:1 in ultrapure type I water (½ Cubic-R1). After incubation (3 min), samples were transferred to a 2 mL Eppendorf tube with pure Cubic-R1 for 24 h incubation on a shaker (60 rpm) at 37 °C. The following day, the samples were washed in 0.1% Triton X-100/TBS for 3 × 15 min on shaker (60 rpm) at RT, transferred to a 2 mL Eppendorf tube with Alexa 555 EdU click-iT reaction cocktail (Click-iT EdU Cell Proliferation Kit for Imaging, Alexa Fluor 555—C10338, Invitrogen), and incubated for 2 h on a shaker (60 rpm) at 37 °C. Afterwards samples were washed in 0.1% Triton X-100/TBS for 3× 15 min on shaker (60 rpm) at RT, and transferred to a new 2 mL Eppendorf tube with Cubic Reagent-2 (Cubic-R2—25% Urea, 50% Sucrose, and 10% Triethanolamine in ultrapure type I water, Sigma-Aldrich) containing Hoechst 33342 (B2261, Sigma-Aldrich) 1:100 dilution, and incubated overnight on shaker (60 rpm) at 37 °C. The next day, the samples were transferred directly to a 2 mL Eppendorf tube with Cubic-R2 and incubated on a shaker (60 rpm) at 37 °C for 48 h until imaging.

### 2.8. Multiphoton Imaging

Imaging of whole hearts was performed on a Multiphoton Microscope Olympus FV-1000 with a UMPlanFL N 10×/0.30 W objective right after the end of CUBIC tissue clearing. No samples were stored long-term following the tissue clearing protocol. Prior to imaging, samples were placed on a glass slide with a Ø 18–22 mm cavity and 1.75 mm depth (Assistent, REF 42415010) containing Cubic-R2; a 25 × 40 mm coverslip was put on the top for imaging and sealed with base coat nail polish. Camera settings were standardized: the number of Z-stacks was optimized according to each heart (120–180 stacks with 8.05 μm steps), Scan mode: Unidirectional, Sequential: Frame, 2.0 μs/pixel, Aspect ratio: 1600 × 1600 pixels, RXD1 (Hoechst): MP 755 nm and 15.8% laser power, RXD3 (EdU): MP 745 nm and 21.8% laser power. Imaris (Bitplane Scientific Software; version 10.0) was used for image analysis and quantification. For quantification of area and volume, a “Surface” was manually created by free-hand drawing the ventricle’s contour in all planes with the slicer rendering tool on. This created surface was used to mask all the channels (Hoechst and EdU) with voxels outside the surface to 0.0, to ensure that only signal inside the created surface would be used for the following spots creation. For nuclei count, “Spots” were created using the masked channels to ensure that only nuclei in the ventricle area were accounted for. Spots-estimated XY diameter was set to 8.0 μm and estimated Z diameter to 24 μm with background subtraction, and threshold above 53 for both EdU and Hoechst channels.

For cell cycle estimation, created Hoechst and EdU spots were quantified by using the distance analysis tool, which estimates colocalization, and set to consider spots at an 8.0 μm distance maximum, thus ensuring a more accurate quantification of EdU^+^ nuclei. Adjustments for the total number of Hoechst nuclei had to be performed considering the following formula: Total Hoechst = Imaris-generated Hoechst spots count + (Imaris-generated EdU spots count − Imaris-generated EdU/Hoechst overlapping spots count).

### 2.9. Cardiomyocyte Preparation for Cell Sorting and Single Cell RNA Sequencing

Seven days after surgery, Mock (*n* = 14), Sham (*n* = 12), or AR (*n* = 11) hearts were dissected as described above and placed in PBS containing 1% BSA (PBS, PBS/1% BSA). The ventricles were then dissected, washed once in RNase-free PBS/1% BSA before enzymatic dissociation with the semiautomatic GentleMACS tissue dissociation system according to the manufacturer’s instructions (Miltenyi Biotec; Neonatal Heart Dissociation Kit 130–098-373). Samples were then processed as described in Bak et al. [38] and pooled based on condition. Staining for fluorescence-activated cell sorting (FACS) was performed using mouse anti-MYH1 (Mouse IgG2b,k; 1:300; MF20-c; DSHB) and donkey anti-mouse IgG Alexa Fluor 488 (1:200; Invitrogen, A21202), and Hoechst 3342 (Sigma, B2261) was added five minutes prior to sorting (FACSAriaIII, BD Biosciences, Herlev, Denmark). Single-cell 3′ RNA libraries were prepared as previously described [38], using Chromium Single Cell 3′ Reagent Kits v3.1 (10X Genomics, Leiden, Netherlands) in combination with a Next Gem G Chip (10X Genomics, 1000120) and Single Cell 3′ Gel Beads (10X Genomics, 1000121). NGS libraries were constructed by adapter ligation and PCR-mediated sample indexing (no. of cycles: 13) using the Single Index Kit T Set A (10X Genomics, 1000213). Libraries were sequenced on the Illumina NovaSeq 6000 platform using NovaSeq 6000 S2 Reagent Kit (100 cycles) V1 (Illumina, Copenhagen, Denmark) (Read 1 = 28 cycles, i7 Index = 8 cycles, Read 2 = 64 cycles).

### 2.10. Single Cell RNA Sequencing Data Analysis

#### 2.10.1. Read Alignment and Construction of Gene Expression Matrix

This was performed as previously described in Bak et al. [38] with some modifications. The generated FASTQ files were aligned to the ENSEMBL *Danio rerio* genome using the splice-aware aligner STAR [44]. Following, STAR [44] used the *Danio rerio* transcriptome reference (GRCz11.98) to segregate the mapped reads as previously described [38].

#### 2.10.2. Clustering and Uniform Manifold Approximation and Projection (UMAP) Visualization

Using the R package Seurat [45] v 4.0.1, quality control was performed for each of the loaded datasets as recently described [46]. Briefly, cells that expressed fewer than 200 genes and cells that did not express the CM-specific marker genes: myl7, myh7l, cmlc1, and tnnt2a were excluded. Dimensionality reduction by principal component analysis (PCA) was performed; subsequently, the PCA data analysis was used as input for visualization by UMAP clustering [47,48]. Cell clustering by expression pattern was performed by first calculating the k-nearest neighbors and constructing the shared nearest neighbor (SNN) and next optimizing the modularity function to determine clusters. Integration was performed using the top 20 principal components. Cell clustering (Louvain algorithm), UMAP, and Heatmap visualization were performed with the same 20 principal components and a resolution of 0.2 (for integrated AR, Sham, Mock) or 0.15 (for integrated AR, Sham). Then differentially expressed genes between clusters of interest with a minimum log fold change of 0.25 were found using FindMarkers. A functional gene analysis was performed by identifying enriched Gene Ontology (GO) terms representing differentially expressed genes, using the ClusterProfiler 4.0 R-packaged [49] and the “org.Dr.eg.db” library with ont = “Biological processes” (“BP”), pAdjustMethod = “Benjamini–Hochberg Procedure” (“BH”), and cutoff values = 0.01. Moreover, when applied, all samples were integrated using FindIntegrationAnchors and IntegrateData, filtered, normalized, and scaled as described above, with generated UMAP plots depicting cell cycle phases, clusters, and original data affiliation for each individual sample.

Cells were assigned to one of three cell cycle stages (G1-, S-, or G2/M-phase) based on canonical cell cycle gene sets [50] using Seurat’s CellCycleScoring function to compute S-phase and G2/M-phase cycling scores for each cell. To obtain a more stringent definition of cycling cells, we classified cells with S and G2/M scores below 0.1 as G1. This threshold was chosen to exclude cells with low cell cycle scores, corresponding to cells near baseline score, i.e., non-cycling G1 cells and the obtained scores were in line with the EdU data.

Zebrafish identified up and downregulated genes were converted to mouse orthologs with Ensembl biomaRt 2.52.0, using drerior_gene_ensemble version 102. Only orthologs with a confidence of 1 were selected.

Single-site analysis was used for prediction of TFs (oPOSSUM version 3.0) [51,52,53], all genes in the current dataset as background, all vertebrate profiles with a minimum specificity of 8 bits, conservation cutoff 0.40, matrix score threshold 85%, up/downstream sequence 5000/5000. In addition, oPOSSUM results were supported by GSEA using the Molecular Signature Database [54,55,56] and transcription factor targets (TFT).

### 2.11. In Situ Hybridization and Immunofluorescence

Hearts for in situ hybridization were dissected, embedded in O.C.T., and cryo-sectioned in 15 µm thick sections as described above and mounted on SuperFrost^®^ Plus microscope slides (Thermo Fisher Scientific, Odense, Denmark). RNA in situ hybridization was performed using the RNAscope^®^ 2.5 HD Reagent Kit-BROWN (cat. no. 322300, ACD). Heart sections were fixed in 10% NBF for 15 min at RT and then dehydrated in increasing concentrations of ethanol (50%, 70%, 100%, 100%), blocked in hydrogen peroxide for 15 min at RT, and rinsed in Milli-Q water (from PURELAB flex system, ELGA VEOLIA, Glostrup, Denmark) before incubation with RNAscope^®^ Protease IV for 15 min at RT. Following, the sections were rinsed twice in PBS and incubated overnight with RNAscope^®^ probe (Dr-anxa2a-C1 (cat. no. 1045311-C1) or Dr-tmsb4x-C1 (cat. no. 1045321-C1)) diluted 1:1 in RNAscope probe diluent (cat. no. 300041) at 40 °C. On the following day, sections were rinsed twice in RNAscope^®^ wash buffer before signal amplification and signal development according to the manufacturer’s instructions for RNAscope^®^ 2.5 HD Reagent Kit-BROWN, however, leaving out amplification steps 5 and 6.

Consequently, sections were rinsed twice in TBS containing 0.05% Tween (TBS+T), blocked in TBS/2% BSA for 20 min at RT, and incubated with mouse anti-MYH1 (Mouse IgG2b,k; 1:200; MF20-c; DSHB) diluted in 1% BSA/TBS for 2 h at RT and rinsed three times in TBS+T. Following, sections were incubated for 90 min at RT with secondary donkey anti-mouse IgG Alexa Fluor 488 (1:200; Invitrogen, A21202) diluted in TBS/1% BSA and rinsed three times in TBS+T afterwards. Before mounting with antifade medium with DAPI (Vectashield, Vector Laboratories, H-1200), the sections were rinsed once in TBS. Images were acquired using either a Zeiss LAM880 microscope (ZEISS, Oberkochen, Germany) or an Olympus FV3000 confocal microscope (Olympus, Tokyo, Japan).

### 2.12. Neonatal Mouse Cultures and Viral Transduction

Neonatal (P0) primary cardiac cultures were established and transduced with adenovirus (Ad) as previously described [38]. Briefly, P0 mouse pups were euthanized by decapitation, hearts were quickly removed, and the ventricles were dissected under a stereomicroscope. Dissected ventricles were pooled in a tube with a cardioplegic buffer (MIB; 1.2 mM KH_2_PO_4_ (pH 7.4); 0.25 g/L Na_2_CO_3_; 6.44 g/L NaCl; 2.6 mM KCl; 1.2 mM Mg_2_SO_4_; 11 mM glucose, Sigma-Aldrich) supplemented with 1% Bovine Serum Albumin (BSA; MIB/1% BSA) before enzymatic dissociation in the GentleMACS tissue dissociator system with heaters as described by the manufacturer (MACS Miltenyi Biotec; Neonatal Heart Dissociation Kit 130-098-373). Dissociated cells were resuspended in growth medium and seeded at a cell density of 410,000 cells/well in 12-well plates pre-coated with extracellular matrix (ECM) in growth medium (79.5% DMEM (Dulbecco’s modified Eagle’s medium, Thermo Fisher, Cat. no. 10566-016), 19.5% Medium 199 (Thermo Fisher, cat. no. 11150-059), and 1% FBS (Lonza) supplemented with 1% penicillin-streptomycin (PS) solution at 37 °C under a humidified atmosphere of 5% CO_2_ 24 h prior virus transduction. Adenoviral constructs were generated using adenoviral human type 5 (dE1/E3) as backbone and a CMV promoter to drive expression of the gene of interest by Vector Biolabs (PA, USA). Ad-GFP-*Anxa2a*, Ad-GFP-*Insm1*, and Ad-GFP-*Tead1* were generated using mouse cDNA (GenBank: BC051240.1 and GenBank: NM_001166585, respectively). GFP, and *Anxa2a*, *Insm1*, or *Tead1* were expressed under separate CMV promoters. Ad-GFP (cat. no. #1060, Vector Biolabs) was used as an empty control. Growth medium containing 10 μM EdU was added together with the virus and replenished every 24 h. All cells for quantitative real-time PCR (qRT-PCR) were replenished with medium without EdU. Cells were either fixed in 2.5% NBF diluted in HBSS/5% FBS containing 1% PS for flow cytometry analysis 96 h post-transduction, fixed in the wells in 10% NBF for immunofluorescence, or the RNA was isolated for qRT-PCR 48 h post-transduction. The transduction efficiency was addressed by immunofluorescence microscopy for GFP during culturing, qRT-PCR (GFP and/or gene of interest), and flow cytometry for GFP. For both setups, 4–6 experiments, each representing CMs from 4–9 P0 hearts, were performed.

### 2.13. qRT-PCR and Flow Cytometry Analysis of P0 Mice Cardiomyocytes

qRT-PCR analyses were performed on P0 mice CMs 48 h post-adenoviral transduction and flow cytometry analyses were performed on cells 96 h post-adenoviral transduction as previously described above and in Bak et al. [38] For the list of primers used in the qRT-PCR analysis, refer to Table S1. Primers annealing temperatures were 57 or 60 °C and 2 ng cDNA was used for each sample. The obtained data were analyzed by normalization to multiple stably expressed endogenous genes according to the qBase Plus 3.2 or 3.4 platform (Biogazelle, Gent, Belgium). For flow cytometry, the antibodies used were mouse anti-MYH1 (1:300, MF20-c, DSHB), rabbit anti-GFP (1:500, ab290, Abcam, Søborg, Denmark), donkey anti-mouse IgG Alexa Fluor 555 (1:200, A31570, Invitrogen), and donkey anti-Rabbit IgG Alexa Fluor 488 (1:200, A21206, Invitrogen).

### 2.14. Statistical Analysis

All statistical analyses were performed using GraphPad Prism version 9.5.0 or newer for Mac OS X (GraphPad Software, San Diego, California, USA, www.graphpad.com). For all the analyses performed, information on the exact value of *n*, mean, and SD can be found in the graph under the Y-axis title and in the figure legend. For time series EdU cell counts, an ordinary two-way ANOVA was performed to account for both variation between technical replicates and biological samples, followed by Fisher’s LSD test with significance set to *p*-value ≤ 0.05. For evaluating the Fold change of EdU^+^ CMs, a Fisher’s exact test was performed with significance set to *p*-value ≤ 0.05. For whole hearts and injury border and remote zone EdU counts, a mixed-effects two-way ANOVA was performed, followed by the two-stage linear step-up procedure of the Benjamini, Krieger and Yekutieli method [57] as a post-hoc test with significance set to adjusted *p*-value ≤ 0.05 (same as 5% false discovery rate (FDR)), when data passed the normality test. In addition, Geisser–Greenhouse correction was also applied. For qRT-PCR data analysis, a two-tailed unpaired or a ratio paired *t*-test was performed with significance set to *p*-value ≤ 0.05, when data passed the normality test.

## 3. Results

### 3.1. Regenerating Ventricular Zebrafish CMs Divide Throughout the Myocardium Peaking Around 7–14 Days After AR

**Figure 1:**
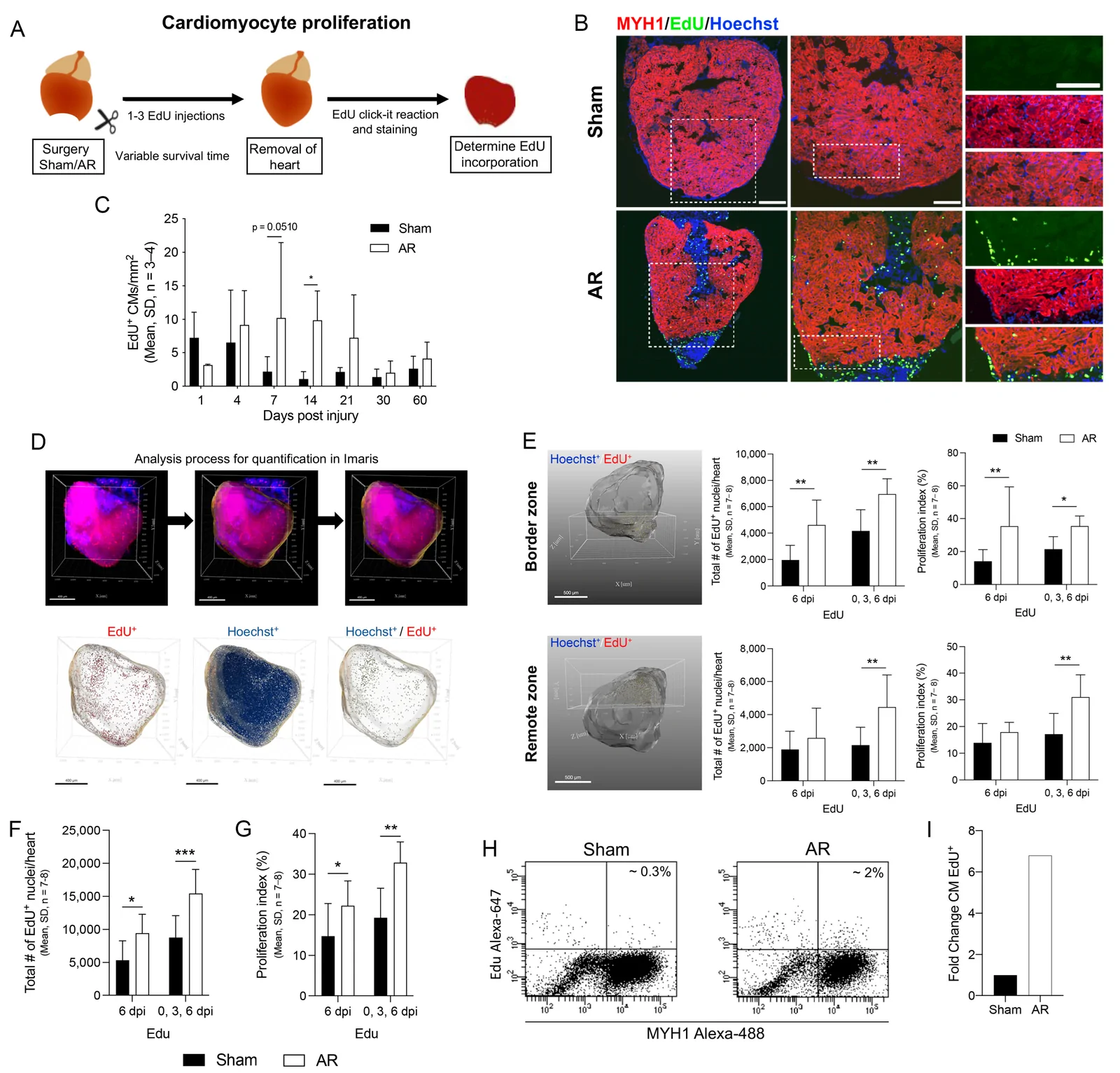
Determination of EdU incorporation after Sham or AR (apex resection) surgery. (**A**), Schematic of study design and workflow. Zebrafish underwent Sham- or apex resection (AR)-surgery and were allowed to recover for 1–60 days post injury (dpi). EdU was injected 1–3 times prior to heart dissection. Following, the EdU-incorporation was determined on zebrafish that had recovered for 1, 4, 7, 14, 21, 30, or 60 dpi by different approaches. (**B**), Immunofluorescence-stained sections from Sham and AR zebrafish at 14 and 4 dpi, respectively (Red: MYH1+ cardiomyocytes (CMs); Green: EdU^+^ cells, Blue: DAPI (nuclei)). Scalebars: left and middle panel: 200 µm, right panel: 400 µm in (**B**); 400 µm in (**D**) and 500 µm in (**E**). (**C**), Percentages of EdU+ CMs/mm^2^ in Sham and AR zebrafish at 1, 4, 7, 14, 21, 30, and 60 dpi (two-way ANOVA using Fisher’s LSD test, * *p*-value ≤ 0.05; data are represented as Mean + SD, *n* = 3–4 sections). (**D**), Representative images of the Imaris analysis process of whole zebrafish hearts showing surface creation for the region of interest and “Spots” creation for EdU^+^, Hoechst^+^, and EdU^+^/Hoechst+ nuclei. (**E**), Representative 3D animation of AR heart, the absolute number of EdU^+^ nuclei, and proliferation index in percentages (Hoechst^+^/EdU^+^) after either 1 or 3 injections with EdU, in the border and remote zone, respectively (data are represented as Mean + SD, *n* = 7–8 hearts per group, * *p*-value ≤ 0.05, ** *p*-value < 0.01). (**F**), Total EdU^+^ nuclei count (two-way ANOVA, adjusted *p*-value ≤ 0.05; data are represented as Mean + SD, *n* = 7–8 hearts per group, * *p*-value ≤ 0.05, *** *p*-value ≤ 0.001). (**G**), Proliferation index in percentage of EdU+ nuclei (two-way ANOVA, adjusted *p*-value ≤ 0.05; data are represented as Mean + SD, *n* = 7–8 hearts per group, * *p*-value ≤ 0.05, ** *p*-value < 0.01). (**H**), Dot plots showing EdU^+^/MYH1^+^ CMs (~0.3% and ~2%, for Sham and AR zebrafish, respectively). (**I**), Graph bars showing the Fold change of EdU^+^ CMs in Sham and AR zebrafish (Fisher’s exact test, *p*-value ≤ 0.0001).

To investigate CM cell cycling in the regenerating zebrafish heart, we performed AR in adult zebrafish (Figure 1A). EdU pulse chasing 24 h before dissection of the hearts (Figure 1A) allowed us to investigate the temporal appearance at 1, 4, 7, 14, 21, 30, and 60 dpi (*n* = 3–4 at each time point) of cycling CMs as defined by EdU^+^ MYH1^+^ in 2D (Figure 1B,C). The number of EdU^+^/MYH1^+^/Hoechst^+^ CMs peaked around 7–14 dpi (Figure 1C). As previously highlighted [13,15,41,58], sham surgery with lesion of the pericardium alone also contributes to CM proliferation, as observed herein at 1 and 4 dpi (Figure 1C). Yet, overall, the number of proliferating CMs was relatively low (Figure 1C), which we speculated could be an obstacle to the subsequent scRNA-seq focusing on regenerating CMs. We, therefore, aimed to evaluate whether some areas of the ventricle contained more proliferating CMs at 7 dpi after AR than others using a modified 3D CUBIC [43] heart clearing approach (Figure 1D–G). Despite comprehensive efforts, the detection of the antigenic determinants of CM markers such as MYH1 failed using the CUBIC approach and we thus instead investigated the overall presence of proliferating cardiac cells throughout the ventricle as defined by EdU^+^/Hoechst^+^ cells. As expected, single (6 dpi) EdU pulse chase labelling revealed a lower number of proliferating cells than repeated (0, 3, 6 dpi) EdU pulsing (Figure 1E–G, *n* = 7–8 hearts in each treatment group and time point) that represent the accumulated number of proliferating cells during the 7 days. However, the number of proliferating cardiac cells still contained, on average, 32.8% of the 7-day accumulated number of proliferating cardiac cells, supporting that 7 dpi is a peaking time point for cell proliferation after AR (Figure 1F), which was suited for our scRNA-seq studies. Spatially, the number of proliferating cardiac cells was more pronounced in the border zone as compared with the remote zone (Figure 1E), but a substantial number of proliferating cells were still dispersed throughout the entire ventricle (Figure 1D,E), suggesting that the entire ventricle was of interest to our scRNA-seq studies. For both whole ventricles as well as the border and remote zones, there was a marked increase in cardiac cell proliferation in AR as compared with Sham (Figure 1E–G and Table S2). These data agree with previous reports [3,4,11,25] and suggest to us that 7 dpi is indeed a key timepoint to study CM proliferation in the entire zebrafish ventricle after AR. Thus, to finally prepare for investigation of regenerating CMs through scRNA-seq, we dissociated 6 dpi EdU-pulsed chased whole ventricles at 7 dpi from both Sham and AR hearts (*n* = 1 pools with 9 hearts in each group) and found by flow cytometry ~2% EdU^+^ CMs in AR-ventricles, whereas Sham represented ~0.3% EdU^+^ CMs (Figure 1H, and Table S2), reflecting approximately 6.8 times more proliferating CMs in AR than in Sham ventricles (Figure 1I). Thus, assuming EdU incorporation indeed reflects mitosis [59,60] in the zebrafish heart with two resulting CM descendants, then around 1% of CMs throughout the ventricle re-enter cell cycling from 6 to 7 dpi after AR.

### 3.2. ScRNA-Seq Based Identification of Genes Defining Regenerating Zebrafish Cardiomyocytes

**Figure 2:**
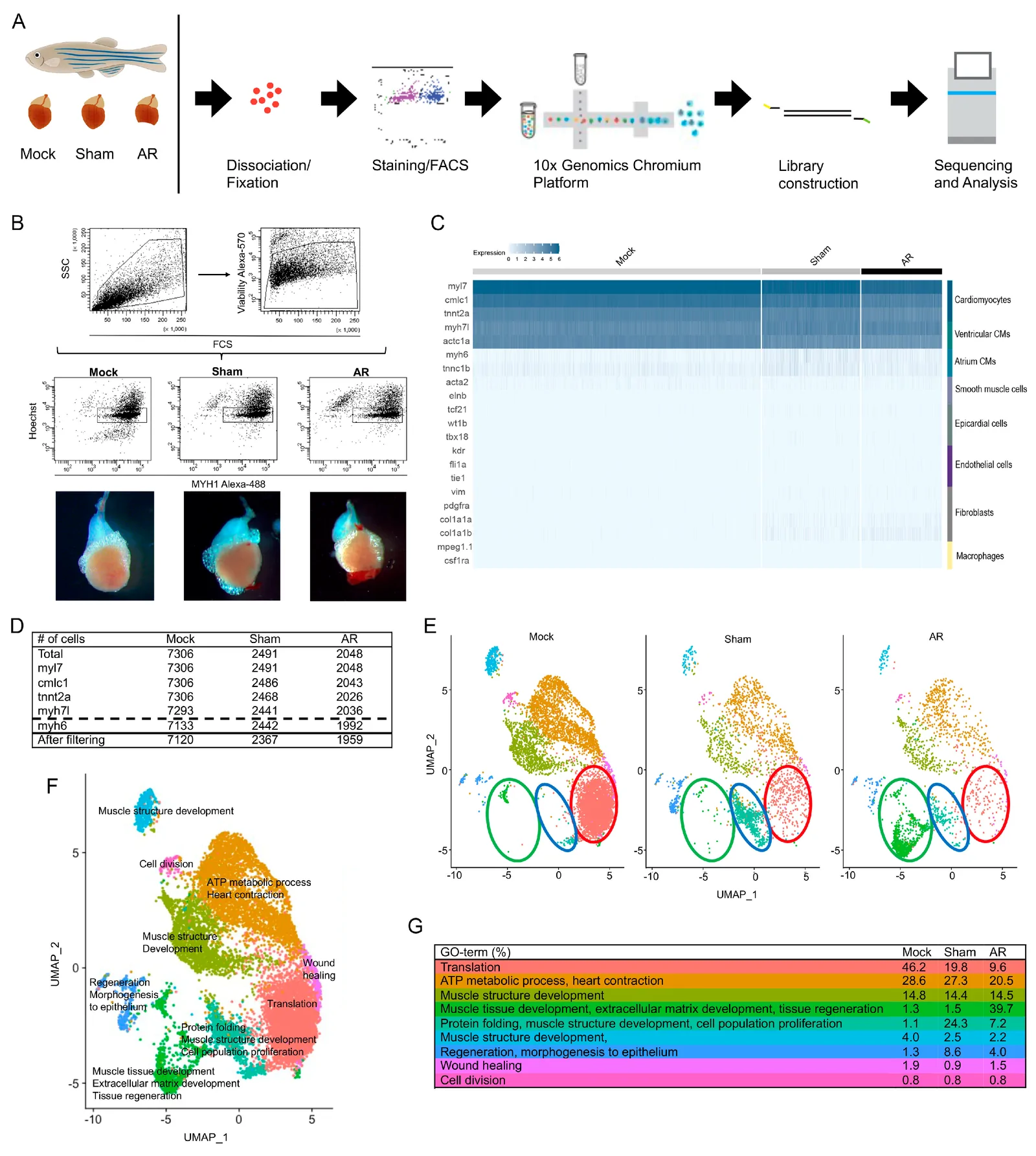
Single-cell RNA analysis of zebrafish hearts. (**A**), Schematic of study design and workflow for setup for scRNA-seq. Hearts were dissected, enzymatically dissociated, and the cells were stained for viability, fixed with methanol, and stained prior to fluorescence-activated cell sorting (FACS) according to the cardiomyocyte (CM) identity of the cells. Sorted cells were further processed for scRNA-seq library generation and subsequent sequencing. (**B**), FACS sorting strategy showing exclusion of apoptotic cells through viability stain and identification of MYH1^+^ CMs and their ploidy. Pictures show representative Mock (*n* = 14), Sham (*n* = 12), and AR (*n* = 11) hearts. (**C**), Heatmap showing high expression of CM-related genes and demonstrating the enrichment in CMs in relation to other cell types. (**D**), Table showing the number of CMs after the filtering strategy and the high purity in CMs in the analyzed samples. (**E**), Uniform Manifold Approximation and Projection (UMAP) plots showing the CM clusters in Mock, Sham, and AR hearts. The shared Mock/Sham/AR cluster is encircled in red, the shared Sham/AR cluster is encircled in blue, and the AR-specific cluster is encircled in green. (**F**), UMAP plot showing the GO terms enriched in the different CM clusters and (**G**), CM percentages in each cluster per condition.

To explore the transcriptomics of regenerating zebrafish CMs at the single-cell level, we performed scRNA-seq as previously described [38] on CMs derived from adult Mock (*n* = 14), Sham (*n* = 12), and AR (*n* = 11) zebrafish ventricles at 7 dpi (Figure 2A). Briefly, dissociated cardiac cells were immediately stained for viability and then fixed to preserve the gene expression profile as close as possible to the in vivo scenario, while maintaining RNA integrity (Figure S1A,B). As recently described [46], we used a stringent approach to validate that potential ambient RNA did not interfere with biological interpretations for samples with relatively low RIN. From our microscopy analysis (Figure 1), we knew that proliferating CMs most likely remain a small subset of the cardiac cells, and we thus performed cell sorting for MYH1^+^ (CM marker) to increase the downstream scRNA-seq resolution for regenerating CMs. Likewise, to avoid too many dropouts in the downstream scRNA-seq analysis, we excluded dead cells using the viability stain added before fixation, but also Hoechst staining to get rid of cell debris (Hoechst^−^) and cell clumps (>4n and >forward scatter threshold) (Figure 2B, Figure S1C). CMs sorted from three pools of zebrafish hearts in each group were pooled (33518 in Mock, 15947 in Sham, 9312 in AR) and used as input for scRNA-seq library generation before sequencing. After scRNA-seq quality checking [46], we used the R package Seurat (4.0.1) [45] and found that 95–97% of the sorted and analyzed cells were ventricular (*myl7*, *cmlc1*, *tnnt2a*, and *myh7l* mRNA positive) zebrafish CMs (Figure 2C,D). After filtering for CMs, data were log-normalized, variable genes identified, and data were scaled prior to clustering and visualization using UMAP plots (Figure 2E). By comparing the UMAPs for Mock, Sham and AR, we identified nine clusters, of which three clusters differed between conditions: (i) an AR-specific cluster, (ii) a shared Sham/AR cluster, and (iii) a shared Mock/Sham/AR cluster (Figure 2E). The percentage of CMs in the three clusters were for the (i) AR specific cluster 1.3%, 1.5%, and 39.7% (encircled in green in Figure 2E); (ii) the shared Sham/AR cluster 1.1%, 24.3%, and 7.2% (encircled in blue in Figure 2E) and for the (iii) shared Mock/Sham/AR cluster 46.2%, 19.8%, and 9.6% (encircled in red in Figure 2E), for Mock, Sham and AR, respectively (Figure 2E–G). Gene Ontology (GO) terms characterizing the AR-specific cluster included “Muscle tissue development”, “Extracellular matrix organization”, and “Tissue regeneration” (Figure 2F,G), whereas the shared Sham/AR cluster was defined by “Protein folding”, “Muscle structure development”, and “Cell population proliferation” (Figure 2F,G). The Mock/Sham/AR cluster represented only a single GO-term “Translation” (Figure 2F,G), and since the percentage of CMs in this cluster was high for Mock, but decreasingly lower for Sham and AR, we do speculate this cluster to be the “origin” of both the AR specific- and the shared Sham/AR clusters (Figure 2G), although we did not perform any pseudotime analysis. Moreover, it seems likely that the Sham/AR shared cluster reflects a general wound response in which CMs are affected by the peri-/epicardial lesion performed in both Sham and AR [41] and that was also seen upon microscopy (Figure 1B,C). The latter agrees with previous reports showing that the peri-/epicardium impacts zebrafish heart regeneration [13,61,62].

For all three conditions, a small “Cell division” CM cluster was characterized (Figure 2F), but since the percentage was equal (0.8%) between conditions, we do not consider these CMs specific to heart regeneration. Indeed, overall cell cycle analysis using G2/M and S cell cycle phase-associated genes showed a blurred picture, where S- and G2/M CMs were distributed throughout most clusters for Sham and AR, whereas for the Mock they were mainly found within the “Cell division” and the shared Mock/Sham/AR clusters (Figure S2B). We did though notice slightly higher percentages of CMs undergoing cell cycling in Sham (2.2% (S) and 3.5% (G2M)) and AR (2.9% (S) and 2.3% (G2M)), as compared with Mock (1.2% (S) and 1.5% (G2M)) (Figure S2A), but whether these percentages are significant remains unknown due to pooling of samples in the analysis.

**Figure 3:**
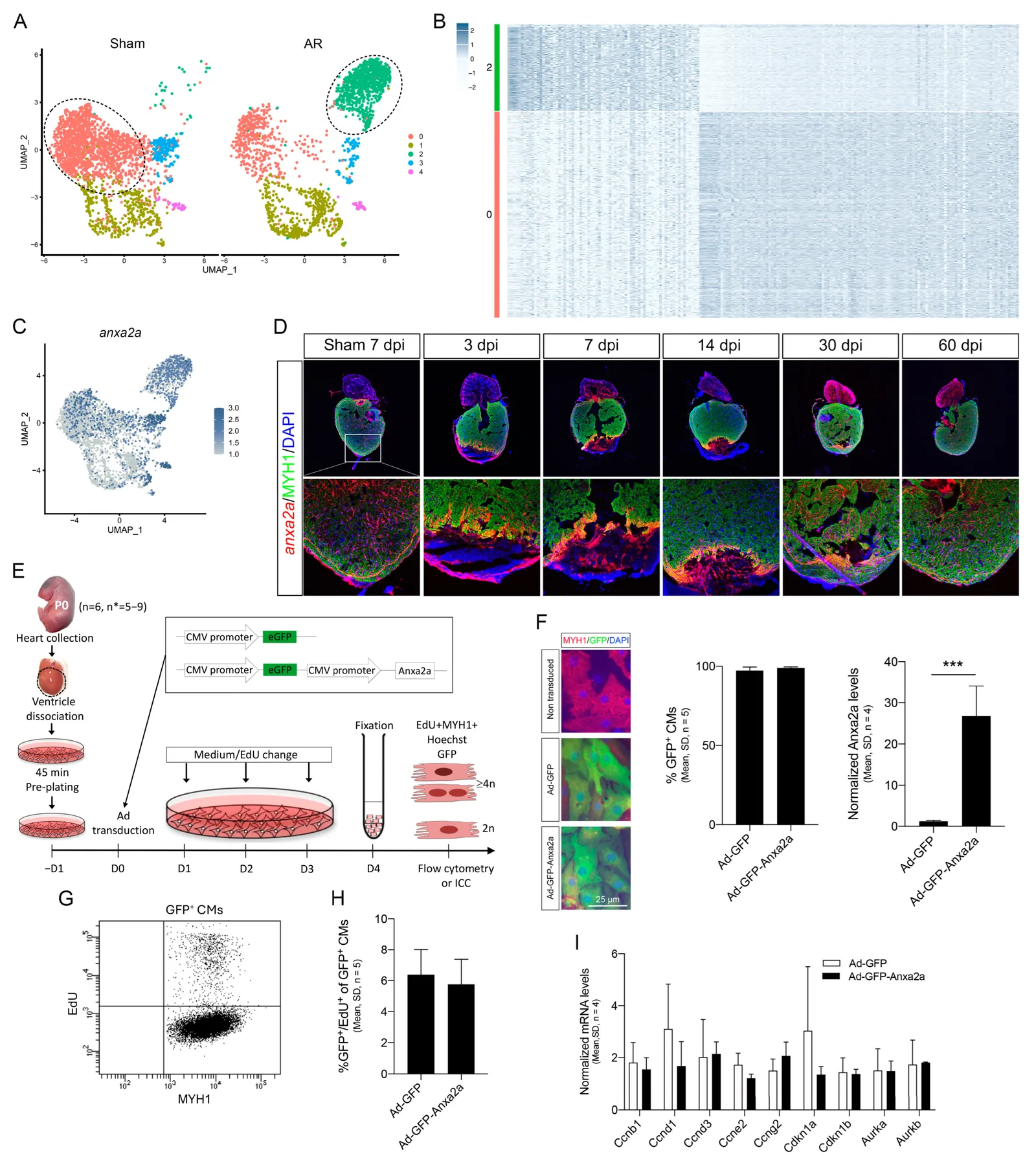
Cluster and transcription factor (TF) analysis after scRNA-seq in zebrafish hearts. (**A**), Uniform Manifold Approximation and Projection (UMAP) plots showing the most prominent clusters in Sham/AR (“0”) and in AR (“2”). (**B**), Heatmap showing differential expression of several genes in the shared Sham/AR cluster (“0”) and in the AR-specific cardiomyocyte (CM) cluster (“2”). (**C**), UMAP plot showing the expression of the upregulated gene anxa2a in AR. (**D**), Representative images from the immunofluorescence in situ analysis showing high expression at 7, and 14 days post-injury (dpi) and localization of *anxa2a* in AR hearts at 3, 7, 14, 30, and 60 dpi compared with the Sham heart. A 7 dpi Sham heart, which is representative of Sham hearts at all time-points, is shown (Red: *anxa2a*^+^ cells; Green: MYH1^+^ CMs; Blue: DAPI (nuclei). (**E**), Schematic of study design and workflow for setup of mouse primary P0 cardiomyocytes (mCMs) and overexpression of Anxa2a using an adenovirus-based transduction strategy. Briefly, for gene overexpression and recognition of the transduced CMs, human adenovirus type5 (dE1/E3) with GFP (CMV promoter) and the desired gene (CMV promoter) (denoted Ad-GFP-Anxa2a or Ad-GFP for the control virus) were used. Primary cultures were set up and 24 h later, virus was added to culture media containing (+) or not containing (−) EdU. Following, culture media +EdU or −EdU was changed every day until 48 h or 96 h after virus transduction, when cells were harvested and fixed for qPCR or flow cytometry analysis, respectively (*n* = 6 with 5–9 (n*) P0 hearts in each experiment). (**F**), Transduction efficiency shown by immunofluorescence of non-transduced, Ad-GFP-, and Ad-GFP-Anxa2x-transduced cells, and bar graphs showing the percentages of GFP^+^ mCMs after flow cytometry at 96h after viral transduction and the qPCR-based mRNA levels of Anxa2a in Ad-GFP or Ad-GFP-Anxa2a mCMs at 48 h after virus transduction (normalized against *Actb* and *B2m* genes; Unpaired *t*-test, *** *p*-value > 0.001; data are represented as Mean, +SD, *n* = 5 biological replicates). (**G**), Representative flow cytometry plot of MYH1 and EdU expression in GFP^+^ mCMs 96 h after transduction with Ad-GPR-Anxa2a. (**H**), Bar graph showing the percentages of EdU^+^ GFP^+^ mCMs 96 h after transduction with Ad-GFP or Ad-GFP-Anxa2a (data are presented as Mean, +SD, *n* = 5 biological replicates). (**I**), Bar graphs showing the qPCR-based mRNA levels of proliferation markers in Ad-GFP or Ad-GFP-Anxa2a mCMs at 48 h after virus transduction (normalized against *Actb* and *B2m* genes; Unpaired *t*-test, data are represented as Mean, +SD, *n* = 4 biological replicates).

Next, to focus on the AR-specific cluster, representing CMs in a transcriptomic regenerative state unique to the AR injury, we excluded the Mock data and merged and integrated Sham and AR CM data into a new dataset (Figure 3A). Here, clustering revealed five distinct clusters (clusters “0–4”) wherefrom an AR-specific CM cluster “2” was easily distinguished (Figure 3A). This cluster of AR-specific CMs further narrowed down the identity of the regenerating CMs and again we speculated that they derived from the shared Sham/AR cluster (“0”) of more mature/inactive, surgery-responsive CMs that was underrepresented in AR (Figure 3A). To investigate this further, we used the Seurat function FindMarkers to identify differentially expressed genes among the two clusters (Figure 3B). Among these genes, we noticed *anxa2a* [63,64] and *tmsb4x* [65], previously associated with fin and heart regeneration, respectively. We, therefore, analyzed their expression in the AR-specific CM cluster “2” (Figure 3C and Figure S3A) and further confirmed in Sham and AR hearts by in situ *hybridization* (Figure 3D and Figure S3B). Both genes were expressed in the regeneration site close to the amputation plane during the first 14 dpi (Figure 3D and Figure S3), validating the scRNA-seq design and data. Where the *tmsb4x* gene is well known in mammalian heart regeneration [65], we instead focused on whether our new knowledge on *anxa2a* could be translated into mammals and whether *Anxa2a* alone could enhance cell cycling of mouse CMs (mCMs) (Figure 3E–H). *Anxa2a* overexpression (CMV promoter) was accomplished using human adenovirus type5 (dE1/E3) with parallel GFP expression (CMV promoter) (Figure 3E, *n* = 6 with 5–9 P0 hearts in each experiment). Transduction efficiency of both *Anxa2a* and the control vector was ~99% as confirmed by immunocytochemistry and flow cytometry (Figure 3F), whereas *Anxa2a* overexpression was confirmed by qPCR (Figure 3F). At 96 h after transduction, the accumulated cell cycle activity in mCMs was quantified by EdU incorporation using flow cytometry for MYH1/GFP/EdU/Hoechst (Figure 3G) and showed that the percentage of GFP^+^/EdU^+^ mCMs was consistent between Ad-GFP and Ad-GFP-*Anxa2a* (Figure 3H). Thus, alone, ANXA2A did not impact mCM cell cycling, which was verified at the mRNA level (Figure 3I).

### 3.3. Predicted Upstream Transcription Factors for Active Regenerating Zebrafish Cardiomyocytes Uncover Potential New Candidates for Enhancing Mammalian CM Cell Cycling

We then speculated if predicted upstream master regulators/TFs affecting entire regeneration gene programs (Figure 3B) in the regenerating zebrafish CMs could be identified by further analysis and comparison of the surgery-responsive cluster “0” and AR-specific cardiomyocyte cluster “2”.

**Figure 4:**
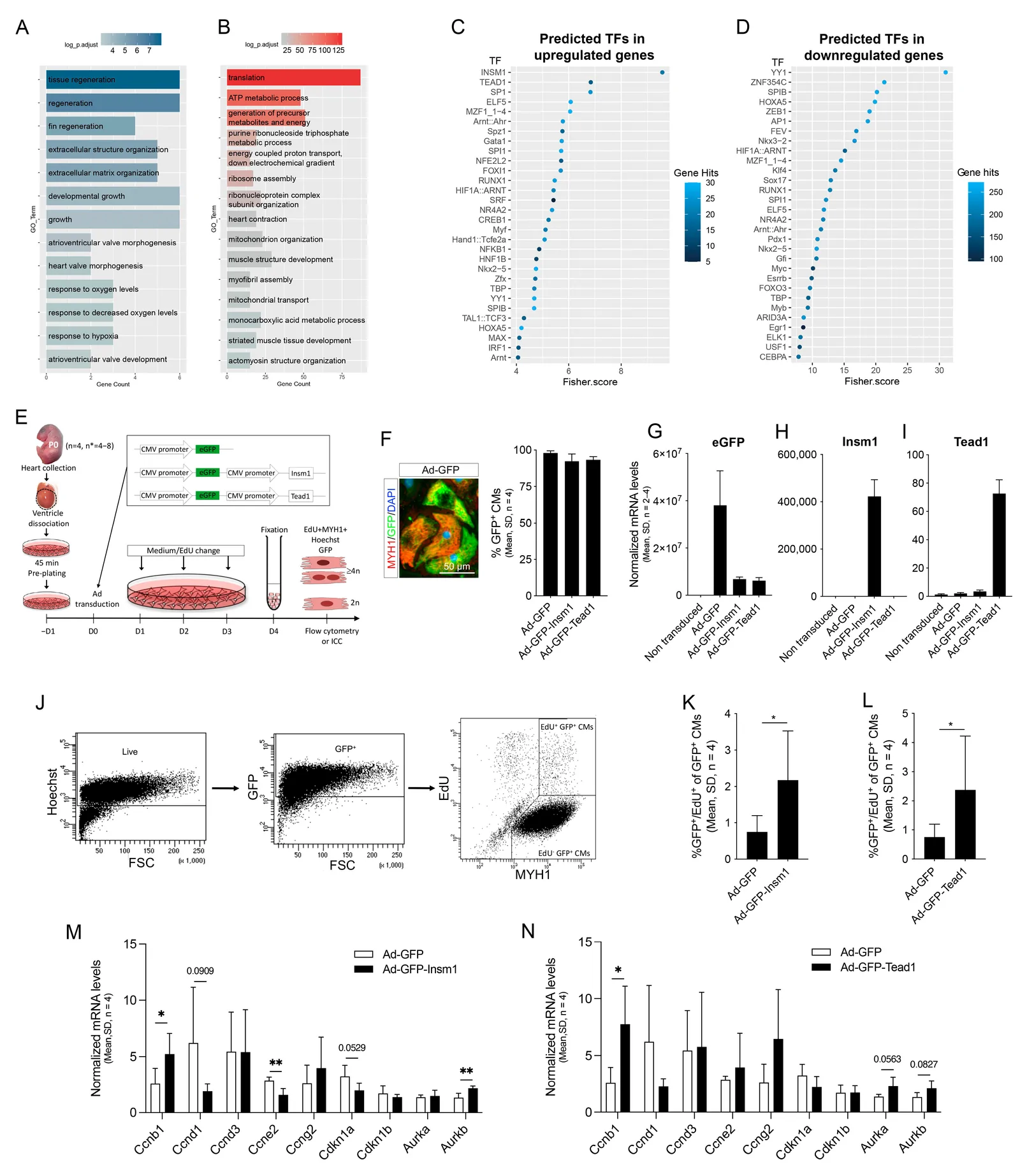
Overexpression of predicted transcription factors (TFs) Insulinoma-associated protein 1 (Insm1) and TEA Domain transcription factor 1 (Tead1) in mouse primary P0 cardiomyocytes (mCMs). (**A**), Bar graphs showing the most enriched GO terms in the upregulated genes in the AR-specific CM cluster (“2”). (**B**), Bar graphs showing the most enriched GO terms in the upregulated genes in the shared Sham/AR cluster (“0”). (**C**), Graph showing predicted TF enrichment after conversion to mouse orthologs using the oPOSSUM platform of the upregulated genes list in the AR-specific CM cluster (“2”). (**D**), Graph showing predicted TF enrichment after conversion to mouse orthologs using the oPOSSUM platform of the downregulated genes list in the AR-specific CM cluster (“2”). (**E**), Schematic of study design and workflow for setup of mouse primary P0 cardiomyocytes (mCMs) and overexpression of *Insm1* and *Tead1* using an adenovirus-based transduction strategy. Briefly, for TF overexpression and recognition of the transduced CMs, human adenovirus type 5 (dE1/E3) with GFP (CMV promoter) and the desired TF (CMV promoter) (denoted Ad-GFP-TF or Ad-GFP for the control virus) were used. Primary cultures were set up and 24 h later, virus was added to culture media containing (+) or not containing (−) EdU. Following, culture media +EdU or −EdU were changed every day until 48 h or 96 h after virus transduction, when cells were harvested and fixed for qPCR or flow cytometry analysis, respectively (*n* = 4 with 4–8 P0 hearts in each experiment). (**F**), Validation in the three Ad-GFP viruses: Bar graphs showing the flow cytometry analysis of GFP^+^ CMs for the transduction efficiency (data are represented as Mean, +SD, *n* = 4 biological replicates), and a representative immunocytochemistry image for GFP expression functionality (Red: MYH1^+^ mCMs; Green: GFP^+^ cells; Blue: DAPI (nuclei)). (**G**–**I**), Bar graphs showing the qPCR-based expression validation of GFP (**G**), *Insm1* (**H**), or *Tead1* (**I**) mRNA levels 48 h after virus transduction (normalized against *Actb* and *Gapdh* genes; data are represented as Mean, +SD, *n* = 4 biological replicates). (**J**), Scatter plots showing the flow cytometry analysis at 96 h after virus transduction for evaluation of the accumulated cell cycle activity in mCMs. (**K**), Bar graphs showing the percentage of GFP^+^/EdU^+^ in Ad-GFP or Ad-GFP-*Insm1* mCMs (Ratio paired *t*-test, * *p*-value ≤ 0.05; data are represented as Mean, +SD, *n* = 4 biological replicates). (**L**), Bar graphs showing the percentage of GFP^+^/EdU^+^ in Ad-GFP or Ad-GFP-*Tead1* mCMs (Ratio paired *t*-test, * *p*-value ≤ 0.05; data are represented as Mean, +SD, *n* = 4 biological replicates). (**M**), Bar graphs showing the qPCR-based mRNA levels of proliferation and dedifferentiation markers in Ad-GFP or Ad-GFP-*Insm1* mCMs at 48 h after virus transduction (normalized against *Actb* and *B2m* genes; Unpaired *t*-test, * *p*-value ≤ 0.05, ** *p*-value > 0.01; data are represented as Mean, +SD, *n* = 4 biological replicates). (**N**), Bar graphs showing the qPCR-based mRNA levels of proliferation markers in Ad-GFP or Ad-GFP-*Tead1* mCMs at 48 h after virus transduction (normalized against *Actb* and *B2m* genes; Unpaired *t*-test, * *p*-value ≤ 0.05; data are represented as Mean, +SD, *n* = 4 biological replicates).

We, therefore, separately subjected up and down regulated differentially expressed genes (Figure 3B, and Tables S3 and S4) to GO term analysis (Figure 4A,B) and observed several GO terms related to regeneration and growth that were upregulated in the AR-specific CM cluster (cluster “2”) (Figure 4A), whereas GO terms related to translation and metabolism as well as heart contraction were enriched for the shared Sham/AR cluster (cluster “0”) (Figure 4B). Foremost, this supported our hypothesis that the AR-specific CM cluster likely represents regenerating CMs that either proliferate or are in an active state supporting heart regeneration. At the time of analysis, we did not recognize a tool developed for upstream gene predictions using zebrafish data. Moreover, to focus on existing mouse orthologs, we instead converted the zebrafish up and downregulated DEG marker genes for the AR-specific CM cluster (cluster “2”) to mouse orthologs and then used the oPOSSUM 3.0 platform for analyzing enrichment of TF-binding sites in their gene orthologs. Accordingly, two different sets of predicted TFs were identified, one for cluster “2” ortholog upregulated genes in AR (Figure 4C) and one for cluster “0” ortholog downregulated genes in AR (Figure 4D). Several predicted TFs were unique for each of the two clusters, whereas other predicted TFs were shared, such as NKX2.5 and YY1, both known to function in cardiac specification [66,67]. Among the top hits for the AR-specific CM cluster (Figure 4C), we identified *insm1* encoding the Insulinoma-associated protein 1, a key marker of neuroendocrine differentiation, but also a target of MESP1 [68], which is known to be a major regulator of cardiogenesis [68,69]. Likewise, the transcriptional repressor TEAD1 (TEA Domain transcription factor 1) was predicted to account for the expression profile in the AR-specific CM cluster. Indeed, TEAD1 is part of the Hippo/YAP signaling pathway that is known to support organ growth, including the heart [70]. To test whether predicted upstream regulators like INSM1 and TEAD1 could enhance CM cell cycling in mammals, we recapitulated our overexpression design from the *Anxa2a* studies (Figure 3E and Figure 4E). Again, transduction efficiencies (*n* = 4 with 4–8 hearts in each experiment) were high (92–98%) as confirmed by flow cytometry and immunocytochemistry (Figure 4F), which was verified by GFP and TF mRNA expression (Figure 4G–I). At 96 h after transduction, the accumulated cell cycle activity in mCMs was visualized by EdU incorporation (Figure S4) and quantified by flow cytometry for MYH1/GFP/EdU/Hoechst (Figure 4J). The percentage of GFP^+^/EdU^+^ CMs significantly increased from 0.8% ± 0.4 (Ad-*GFP*—control) to 2.1% ± 1.4 (Ad-GFP-*Insm1*) and 2.4% ± 1.9 (Ad-GFP-*Tead1*) (mean, SD, *n* = 4) (Figure 4K,L), respectively. Despite this representing a modest increase, it reflects more than a doubling of mCMs undergoing DNA replication upon *Insm1-* and *Tead1* overexpression. To evaluate whether the replicated mCMs were undergoing endoreplication or true division [71,72,73], we performed qRT-PCR at 48 h following transduction (Figure 4M,N). Ad-GFP-*Insm1* mCMs presented a significant increase in *Ccnb1* (Cyclin B1) and *Aurkb* (Aurora kinase B), two factors associated with division in the G2/M phases, while levels of *Ccne2* (Cyclin E2), related to endoreplication [74,75], were significantly decreased (Figure 4M). Moreover, the cell cycle inhibitor *Cdkn1a* (Cyclin-dependent kinase inhibitor 1a, p21) was nominally decreased in Ad-GFP-*Insm1* mCMs as compared with Ad-GFP mCMs (Figure 4M). Similar results were obtained for Ad-GFP-*Tead1* overexpressing mCMs (*n* = 4) (Figure 4N) and suggest that both INSM1 and TEAD1, in at least a small fraction of postnatal mCMs, may override the normal developmental process of terminal differentiation and stimulate mCM cell cycling.

## 4. Discussion

Here, we report a detailed scRNA-seq analysis with prediction of upstream master regulators for regenerating CMs in the injured zebrafish heart, which in proof-of-concept studies enhance cell cycling in mCMs. This testifies to the general idea of translating zebrafish biology to benefit heart repair in humans after MI.

Several methods using 2D analysis have previously been used to quantitate zebrafish CM proliferation with diverging results [58], whereas in-depth knowledge in 3D seems restricted to other measures such as function and tissue architecture [76,77]. Here we used a modified 3D CUBIC protocol to better localize and quantitate the distribution of proliferating cells in the regenerating zebrafish heart to prepare for our scRNA-seq design, but in the future, this approach should allow CM specificity and hence may be valuable for more objective quantification of CM proliferation in the zebrafish heart [58]. Even so, we confirmed in 3D that although the number of proliferating cells was more pronounced in the vicinity of the heart damage, remote areas exhibited cell proliferation as well. Although these remote and injury-border localized CMs may represent distinct transcriptomic profiles, we decided to embrace them all in our scRNA-seq design using fixed zebrafish ventricular CMs. The obtained scRNA-seq data testified to the design being focused on CMs and allowed us to identify a cell cluster belonging exclusively to the zebrafish AR heart, which consisted of CMs with a transcriptomic regenerative phenotype. The existence of a cluster of AR-specific CMs seems in line with previous studies identifying clusters of proliferating CMs specific to cryoinjury [28] or genetic ablation [33]. Integration of our dataset with those previously published for regenerating CMs may be valuable to identify whether regenerating CMs exhibit transcriptomic profiles specific to the injury type, as previously suggested at the single gene level [35].

In the set of differentially expressed genes defining regenerating zebrafish CMs, we clearly identified *anxa2a* as a gene expressed in the vicinity of the injury, which indicates that this gene is important for regenerating CMs. Previously, *anxa2a* has been associated with caudal fin regeneration in zebrafish [63,64]. However, we did not observe any effect on CM cell cycling when translating this new *anxa2a* knowledge into mammals, as exemplified by mCMs. Obviously, this may simply reflect that *anxa2a* is important for another biological process [78] other than CM cell cycling, but it may also reflect that one gene alone has a limited effect on CM regeneration as previously suggested [38]. Indeed, our study suggests that predicted upstream TFs regulating several genes or entire gene programs may be more potent in dictating a regeneration program, as reported previously for other TFs [28,37,79]. In general, many TFs have been recognized as important factors for regulation of CM cellular and subcellular physiology, including cell cycling, karyokinesis, and cytokinesis, and have been applied for CM regeneration-inducing therapies [80]. In our scRNA-seq analysis, we identified a predicted network of TFs, including Tead1 and Insm1, as part of the transcriptomic profile of the regenerating zebrafish CM cluster. Tead1 is a well-known member of the Hippo pathway [70] and has been extensively studied in cardiac development and regeneration [70,81,82,83,84,85,86,87], including CM proliferation [88]. Thus, our Tead1 data are in line with previous literature data [82,84,88], but also verify our scRNA-seq design. Insm1, on the other hand, is a zinc finger TF [89] that has been reported to play important roles in the development, differentiation, and proliferation of several different cell types [90,91,92,93,94,95], but seems not previously associated with CM cell cycling or heart biology. In our proof-of-concept mouse in vitro studies, overexpressing *Insm1* induced a doubling in the number of EdU^+^ mCMs to the same level as in the *Tead1* study. Moreover, this was accompanied by an increase in the levels of the G2/M phase markers CCNB1 and AURKB, which stimulate mCM cell cycling when overexpressed together with CDK1 (*Cyclin Dependent Kinase 1*) [96]. In parallel, Insm1-overexpressing mCMs also showed a decrease in Ccne2 expression, a factor associated with endoreplication [74,75]. This may suggest that INSM1 contributes to mCM cell division rather than endoreplication. Still, the level of mCM cell cycling was modest by overexpressing Insm1 alone and must reflect either that only a small fraction of the neonatal mCMs is able to override the normal developmental process of terminal differentiation or simply that other factors are required as well. This is a scenario often described for many identified factors and beautifully reflects the challenge of mammalian CMs being inert to cell division in the postnatal heart [97,98]. Yet, despite the doubling of EdU-positive mCMs is remarkable it may be insufficient to suggest a general biological change in phenotype. For instance, since the TFs are merely predicted, it could be that neither INSM1, nor TEAD1 are functionally active and/or biologically relevant players in the adult heart. Nonetheless, these predicted TFs may still be interesting to manipulate, seen from a clinical perspective. Currently, we can only speculate on which factors may act in synergy with *Insm1* to enhance CM cell cycling more efficiently. In fact, the entire array of predicted TFs herein may be needed in addition to many others, and we can only speculate at this stage. Moreover, the use of the newer bioinformatic tools for predicting upstream TFs in zebrafish data may be better suited for this task and may likely define other important TFs for regenerating zebrafish CMs but possibly not immediately identify mouse orthologs as aimed for herein using the ortholog conversion strategy. There is also the possibility that regulatory elements are not perfectly conserved across species and that the TF binding site enrichment does not establish direct binding or causality. It is also important to keep in mind that the predicted TF networks are based on a proposed relationship between clusters without further supportive trajectory- or pseudo-time analyses. In addition, the role of the predicted TFs in zebrafish heart regeneration in the AR-specific CM cluster has not been functionally validated in the zebrafish regeneration model; hence, their role in defining and/or regulating regenerating CMs in zebrafish is not established and, therefore, speculative. Finally, it is also likely that regeneration programs differ across injury paradigms, which may limit generalizability to other injury contexts and to mammals. Despite these limitations, we consider the provided approach and resulting scRNA-seq dataset as a potential novel source for further exploration of cell cycling/proliferation/regenerating-stimulating signals in the zebrafish heart, which in the long run may lead to new therapies for MI patients through forced CM proliferation.

## 5. Conclusions

In summary, our study provides a new scRNA-seq dataset and a network of TFs predicted to regulate an AR-specific CM population in zebrafish that may be further explored to unravel zebrafish heart regeneration and eventually utilized for inducing CM proliferation in the mammalian heart.

## Data Availability

The single-cell RNA-seq data have been deposited at Gene Expression Omnibus (GEO) and are publicly available as of the date of publication. Accession number: GSE280871.

## Supplementary Materials

The following supporting information can be downloaded at: https://www.mdpi.com/article/10.3390/cells15161455/s1, Figure S1: RNA integrity number (RIN) and histograms from cell sorting prior to scRNA-Seq; Figure S2: Cell cycle analysis of scRNA-seq data; Figure S3: In situ hybridization of *tmsb4x* in zebrafish hearts; Figure S4: Immunocytochemistry representative images of the accumulated cell cycle activity in mouse cardiomyocytes (mCMs) at 96 h after virus transduction; Table S1: Mouse primer list for qRT-PCR analysis; Table S2: MF20 (MYH1) and EdU^+^ cell counts in Sham and Apex Resected (AR) operated zebrafish hearts; Table S3: Cluster 2 markers down-regulated (cluster 2 vs cluster 0); Table S4: Cluster 2 markers up-regulated (cluster 2 vs cluster 0).

## Abbreviations

The following abbreviations are used in this manuscript:

Ad	adenovirus
AR	apex resection
Aurkb	aurora kinase B
BH	Benjamini–Hochberg procedure
BP	biological processes
BSA	bovine serum albumin
Ccnb1	cyclin B1
Ccne2	cyclin E2
CDK1	cyclin-dependent kinase 1
Cdkn1a	cyclin-dependent kinase inhibitor 1a
CM	cardiomyocyte
CMs	cardiomyocytes
CUBIC	clear, unobstructed brain/body imaging cocktails
Cubic-R1	cubic reagent-1
Cubic-R2	cubic reagent-2
DMEM	Dulbecco’s modified Eagle’s medium
dpi	days post injury
ECM	extracellular matrix ECM
EdU	5-ethynyl-2′-deoxyuridine
FBS	fetal bovine serum
FDR	false discovery rate
GFP	green fluorescence protein
GO	gene ontology
HBSS	Hank’s buffered saline solution
Insm1	insulinoma-associated protein 1
mCMs	mouse cardiomyocytes
MI	myocardial infarction
MYH1	myosin heavy chain 1
NBF	neutral buffered formalin
PBS	phosphate buffer saline
PCA	principal component analysis
PFA	paraformaldehyde
PS	penicillin-streptomycin
qRT-PCR	quantitative real-time PCR
scRNA-seq	single cell RNA sequencing
SNN	shared nearest neighbor
TBS	tris-buffered saline
TBS+T	TBS containing 0.05% Tween
Tead1	TEA Domain transcription factor 1
TFs	transcription factors
TFT	transcription factor targets
UMAP	uniform manifold approximation and projection
