# Supplementary material for: Identifying Transcription Factors Distinguishing Regenerating Cardiomyocytes in the Zebrafish Heart Using Single-Cell Transcriptomics
Source: Cells. 2026 Aug 13;15(16):1455. doi: 10.3390/cells15161455 (PMC13511067; doi:10.3390/cells15161455)
Supplement: Supplementary file 1 [file cells-15-01455-s001.zip › cells-4369929-supplementary.pdf]

SUPPLEMENTARY MATERIAL

SUPPLEMENTARY FIGURES

SUPPLEMENTARY FIGURE S1

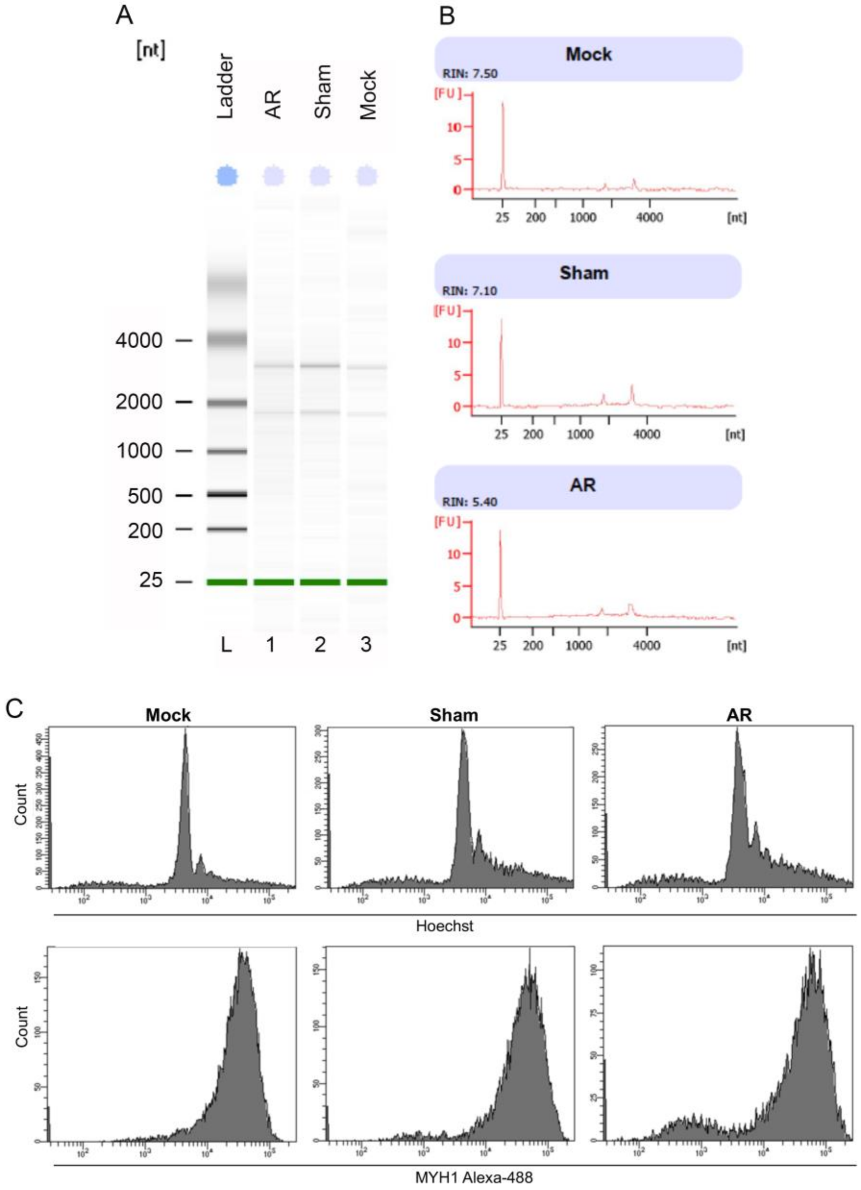

Supplementary Figure S1. RNA integrity number (RIN) and histograms from cell sorting prior to scRNA-Seq**.** RIN measurements post-FACS sorting of Apex resected (AR), Sham operated and unoperated zebrafish heart samples. RIN classes ranges from 1 (degraded) to 10 (intact), thus the tested zebrafish samples have intermediate to good RNA quality. We **A,** Gel-like image showing the patterns of Ladder, AR, Sham, and unoperated zebrafish samples (from left to right). **B,** Electropherograms of the AR, Sham, and unoperated zebrafish samples (from left to right) displaying their RIN measure in the low left corner. C, Histograms for Hoechst (upper panel) and Myh1 (lower panel) from cell sorting prior to scRNA-seq. FU = fluorescence units, nt = nucleotide, AR = apex resection.

SUPPLEMENTARY FIGURE S2

A

	Mock	Sham	AR
G1	97.3	94.3	94.8
G2/M	1.5	3.5	2.3
S	1.2	2.2	2.9
Total	100.0	100.0	100.0

B

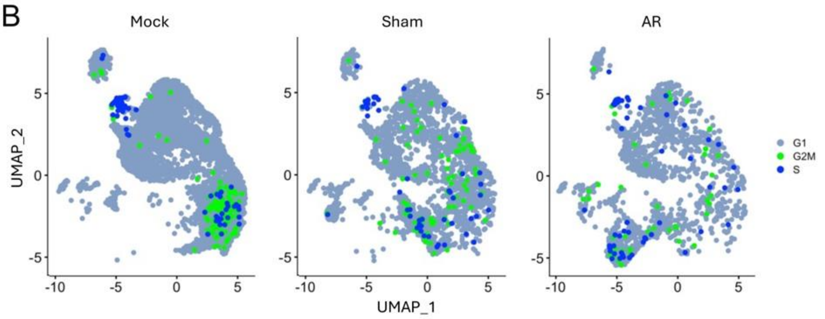

Supplementary Figure S2. Cell cycle analysis of scRNA-seq data. **A,** Table showing percentages of CMs in the different cell cycle phases and conditions. **B,** UMAP plots showing cell cycle phase analysis for Mock, Sham, and AR hearts.

SUPPLEMENTARY FIGURE S3

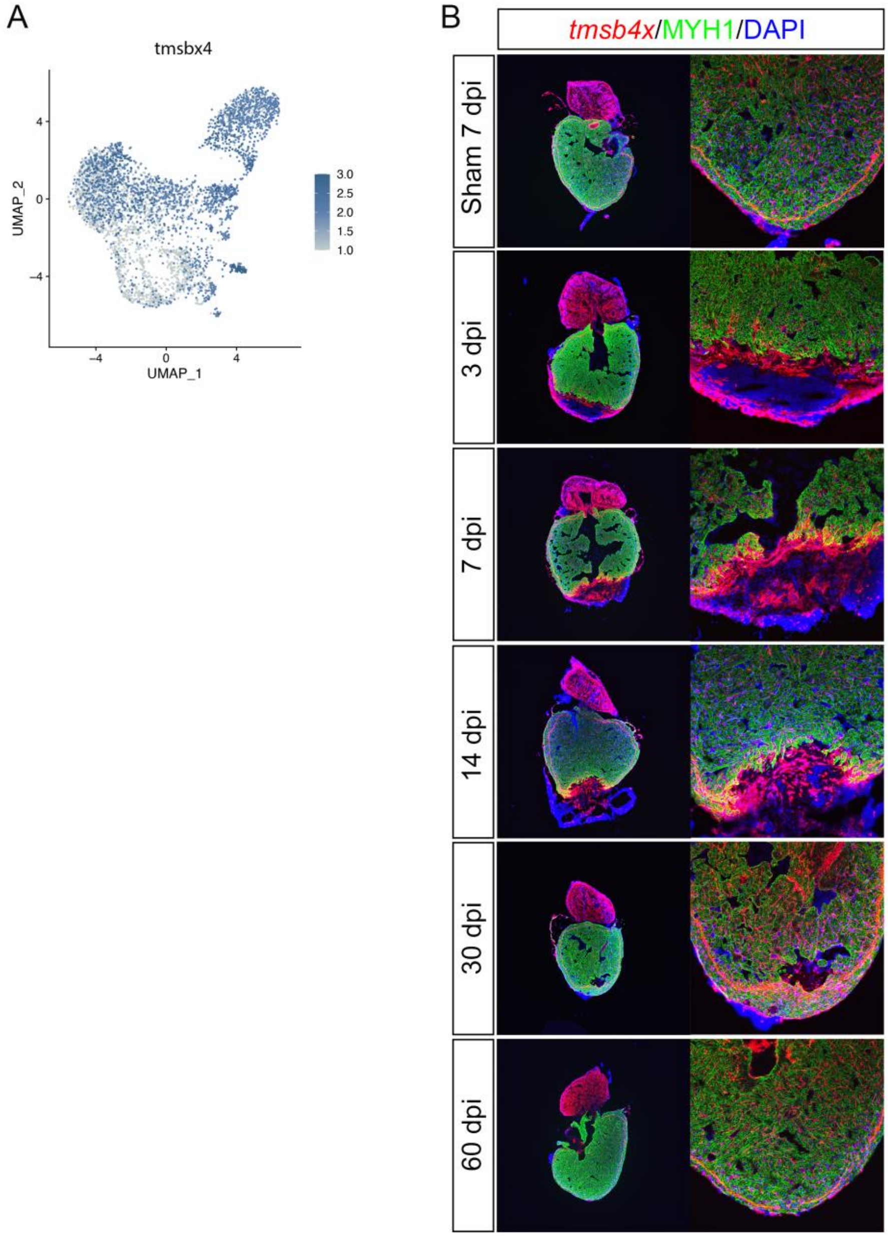

Supplementary Figure S3. *In situ* hybridization of *tmsb4x* in zebrafish hearts. **A**, Uniform Manifold Approximation and Projection (UMAP) plot showing the expression of the upregulated genes in AR, *tmsb4x*. **B**, Immunofluorescence *in situ* analysis showing high expression and localization around the scar site of *tmsb4x* mRNA in Sham and Apex resected (AR) zebrafish hearts at 3, 7, 14, 30, and 60 dpi. A 7 dpi Sham heart, which is representative for Sham hearts at all time-points is shown (Red: $tmsb4x^+$ cells; Green: $myh1^+$ CMs; Blue: DAPI (nuclei)).

SUPPLEMENTARY FIGURE S4

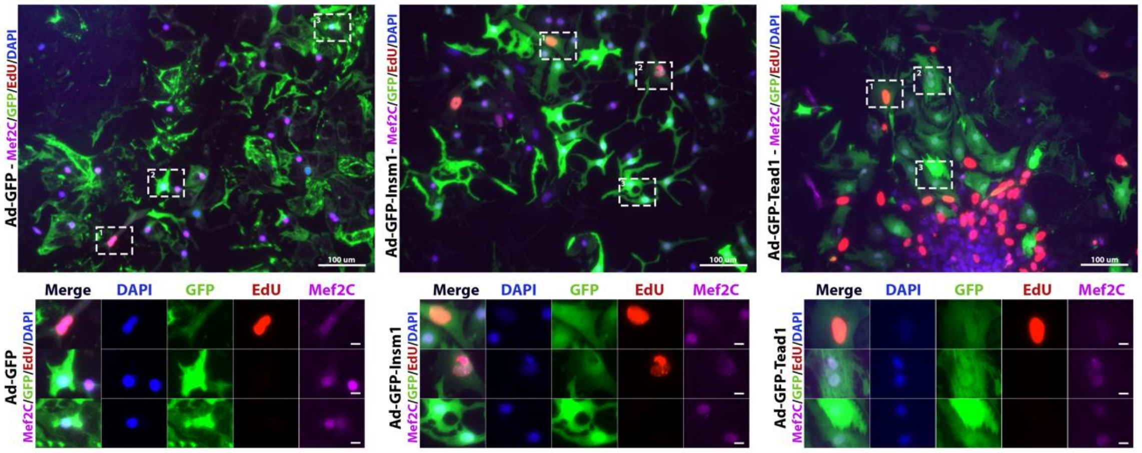

Supplementary Figure S4. Immunocytochemistry representative images of the accumulated cell cycle activity in mouse cardiomyocytes (mCMs) at 96 h after virus transduction. Left panel: Ad-GFP transduced mCMs. Middle panel: Ad-GFP-Insm1 transduced mCMs. Right panel: Ad-GFP-Tead1 transduced mCMs. (Purple: MEF2C^{+} mCM; Green: GFP^{+} cells; Red: EdU^{+} cells; Blue: DAPI (nuclei) Scalebars: low magnifications: 100 µm; high magnifications: 20 µm).

SUPPLEMENTARY TABLES

SUPPLEMENTARY TABLE S1

Supplementary Table S1. Mouse primer list for qRT-PCR analysis.

Gene name	Forward sequence	Reverse sequence
ACTB	GCTGTATTCCCCTCCATCGTG	CACGGTTGGCCTTAGGGTTCAG
B2M	ATGGCTCGCTCGGTGACCCT	TTCTCCGGTGGGTGGCGTGA
GAPDH	GTCGGTGTGAACGGATTTGGC	TGAAGGGGTCGTTGATGGCA
eGFP	GCACAAGCTGGAGTACAACTA	TGTTGTGGCGGATCTTGAA
Insm1	GGAGTTCTAACGAGATGCTGTAG	AGCCCTACCTCAACAAAGATG
Tead1	GTATGCGAGGTTCGAGAATG	ATTTCTCTGGTAGGTGTTTGAG
Ccnb1	GGTGTAACGGCCATGTTTATTG	GGAGAGCTCCATGAGGTATTTG
Ccnd1	GAAGTGGAGACCATCCGCCGC	CCTCACAGACCTCCAGCATCCAGG
Ccnd3	TGTGGCTCCATGGCAGTTGCG	AAGTCGTGCGCAATCACGGCA
Ccne2	TGGCCACCTGTACTGTCTGGAGG	ACCTCCTGTGAACATGCCCAGCTTA
Ccng2	CGACACGATGAAGGATTTGGGGGC	CTCCGGGGTAGCCTCCATCAAGATC
Cdkn1a	ATCCAGACATTCAGAGCCACAGGCA	GCATCGCAATCACGGCGCAA
Cdkn1b	AGTCAGCGCAAGTGGAATTT	AGTAGAACTCGGGCAAGCTG
Aurka	CCTCAGGCTGTATGGCTATTT	GCAATCTTCAACTCTCCGTTTG
Aurkb	ATCCCAACATCCTTCAACTCTAC	GCAGGTTCTCCGGCTTTAT

SUPPLEMENTARY TABLE S2

Supplementary Table S2. MF20 (MYH1) and EdU^+ cell counts in Sham and Apex Resected (AR) operated zebrafish hearts.

	**$MF20^-$ EdU^-**	**$MF20^-$ EdU^+**	**Total NMs**	**$MF20^+$ EdU^-**	**$MF20^+$ EdU^+**	**Total CMs**	**Total viable cells**	**$\%MF20^-$ EDU^+**	**$\%MF20^+$ EDU^+**
Sham	3552	65	3617	11084	35	11119	14736	1.7971	0.3148
AR	3341	138	3479	5748	124	5872	9351	3.9667	2.1117
Fisher Exact Test		$p =$ 0.0001			$p =$ 0.0001				

NMs = Non-Myocytes ($MF20^-$), CMs = Cardiomyocytes ($MF20^+$).

SUPPLEMENTARY TABLE S3

Supplemental Table S3. Cluster 2 markers down-regulated (cluster 2 vs cluster 0).

Gene_Symbol	avg_log2FC	avg_FC	p_val	p_val_adj	pct.1	pct.2
slc25a5	-0.95718699	0.515060215	1.1442E-256	2.0076E-252	1	1
cmlc1	-1.357140494	0.390355231	5.4236E-242	9.5162E-238	1	1
myl7	-0.706451204	0.612825739	1.1124E-236	1.9519E-232	1	1
mt-cyb	-1.13487188	0.455375355	5.9214E-226	1.039E-221	0.998	0.999
mt-co2	-0.882079364	0.542584837	3.2234E-220	5.6557E-216	1	1
mt-co3	-0.901557772	0.535308412	7.7636E-213	1.3622E-208	1	1
mt-atp6	-0.947009637	0.518706504	1.7459E-204	3.0633E-200	0.999	1
tnnt2a	-0.804522937	0.572551377	2.7953E-172	4.9046E-168	1	1
CR383676.2	-1.144164292	0.452451704	1.4272E-169	2.5041E-165	1	0.994
eno3	-1.03130283	0.489268114	1.5223E-110	2.6709E-106	0.934	0.91
atp5mc3b	-0.853924485	0.553277636	2.8858E-106	5.0635E-102	0.989	0.975
atp5f1b	-1.000669496	0.499768024	3.2014E-105	5.6171E-101	0.965	0.945
myh7l	-0.514774216	0.69990246	3.28407E-93	5.76223E-89	1	1
mdh2	-0.866136386	0.548614103	3.34389E-82	5.86719E-78	0.92	0.894
ak1	-0.876259895	0.544777908	9.75967E-77	1.71243E-72	0.92	0.881
tnnc1a	-0.63207847	0.645246148	1.14292E-74	2.00537E-70	0.982	0.969
atp5pd	-0.997834873	0.500750939	5.31891E-67	9.33255E-63	0.87	0.833
pln	-0.740703135	0.598447612	6.61819E-67	1.16123E-62	0.949	0.896
mt-nd1	-0.789082861	0.578711869	1.01335E-66	1.77802E-62	0.925	0.869
ndufa4l	-0.719868867	0.607152626	3.38513E-65	5.93955E-61	0.975	0.942
cox4i1	-0.879580187	0.54352557	2.77639E-62	4.87146E-58	0.928	0.859
ybx1	-0.896798878	0.537077103	2.24382E-61	3.937E-57	0.878	0.82
rpl17	-0.856150949	0.552424439	5.08472E-60	8.92165E-56	0.903	0.84
rpl7a	-0.722608403	0.606000797	3.05417E-55	5.35884E-51	0.937	0.868
rps5	-0.836189999	0.560120836	2.74259E-54	4.81215E-50	0.876	0.814
atp5f1d	-1.10216614	0.465816568	1.13056E-53	1.98368E-49	0.76	0.737
tpm4a	-0.378832941	0.769059465	7.09801E-51	1.24542E-46	0.999	0.993
fabp3	-0.747634389	0.595579339	5.95755E-49	1.04531E-44	0.894	0.828
cox6b2	-0.816854802	0.567678181	7.16439E-49	1.25706E-44	0.893	0.812
idh2	-0.632670967	0.644981208	5.75455E-46	1.00969E-41	0.942	0.891
cox6a2	-0.48467809	0.714656514	1.72329E-45	3.02368E-41	0.992	0.963
slc25a3b	-0.834826714	0.560650376	1.32239E-44	2.32027E-40	0.851	0.772
cox5aa	-0.880975463	0.543000163	6.59317E-38	1.15684E-33	0.821	0.758
rps3a	-0.621505136	0.649992449	2.90644E-36	5.09964E-32	0.924	0.828
rpl19	-0.667242298	0.629709222	3.6024E-36	6.32078E-32	0.926	0.824
mdh1aa	-0.873754015	0.545724979	1.03604E-35	1.81783E-31	0.779	0.703
atp5l	-0.624637977	0.648582509	1.49738E-35	2.6273E-31	0.933	0.843
vdac2	-0.762922731	0.589301265	3.97073E-35	6.96704E-31	0.86	0.774
rps27a	-0.701744044	0.614828504	3.81133E-34	6.68736E-30	0.881	0.781
rpl13	-0.703795323	0.613954938	2.91594E-33	5.11632E-29	0.877	0.788
atp5pf	-0.748778099	0.595107375	8.67285E-33	1.52174E-28	0.818	0.746
atp5fa1	-0.660542755	0.632640247	6.60354E-32	1.15866E-27	0.891	0.805
atp5po	-0.653269697	0.635837629	1.95705E-31	3.43384E-27	0.888	0.794
ldhba	-0.709055757	0.611720378	2.5116E-31	4.40686E-27	0.908	0.795
cox5b2	-0.671288051	0.627945801	2.93707E-30	5.15339E-26	0.911	0.801
atp5mc1	-0.794573125	0.576513731	3.2839E-29	5.76194E-25	0.906	0.79
eef1a1l1	-0.388538162	0.763903252	8.26174E-29	1.4496E-24	0.993	0.965
rpl28	-0.770339164	0.586279629	1.33923E-28	2.34982E-24	0.768	0.71
rps7	-0.721177047	0.606602334	1.86674E-28	3.27538E-24	0.864	0.767

acta1b	-0.46893162	0.722499443	4.07761E-28	7.15457E-24	0.983	0.945
tnni4a	-0.563918168	0.676462484	6.79147E-28	1.19163E-23	0.931	0.819
rplp1	-0.462889167	0.72553184	6.30294E-27	1.10591E-22	0.948	0.865
rps4x	-0.724846425	0.60506145	7.03137E-26	1.23372E-21	0.825	0.722
atp5pb	-0.679141546	0.624536785	1.19467E-25	2.09617E-21	0.857	0.756
gapdh	-0.276351736	0.825676337	1.51856E-25	2.66446E-21	1	0.996
rplp0	-0.528474251	0.693287545	3.00812E-25	5.27805E-21	0.926	0.828
ndufb7	-0.853108143	0.553590794	4.94126E-25	8.66993E-21	0.708	0.651
mb	-0.338784341	0.790707305	8.26664E-25	1.45047E-20	1	1
atp5if1b	-0.92136903	0.528007735	5.89624E-24	1.03455E-19	0.683	0.639
rpl24	-0.644232962	0.63983288	1.54162E-23	2.70493E-19	0.865	0.749
rpl13a	-0.667325176	0.629673049	3.52588E-22	6.18651E-18	0.813	0.715
rpsa	-0.518156243	0.698263641	7.89929E-22	1.38601E-17	0.914	0.799
rpl21	-0.583056768	0.667547884	2.86516E-20	5.02721E-16	0.857	0.743
ckma	-0.340819911	0.789592444	1.2655E-19	2.22044E-15	0.989	0.957
atp2a2a	-0.582452276	0.667827646	7.50491E-19	1.31681E-14	0.801	0.696
nme2b.2	-0.565959291	0.675506103	2.02043E-18	3.54504E-14	0.932	0.807
rps14	-0.497885469	0.708143934	2.72033E-18	4.77308E-14	0.9	0.776
rpl23	-0.522347888	0.696237831	3.83876E-18	6.73549E-14	0.885	0.753
rps13	-0.611776944	0.654390204	8.20555E-17	1.43975E-12	0.834	0.71
rps19	-0.460981507	0.726491837	9.57035E-17	1.67921E-12	0.9	0.78
mt-co1	-0.262937802	0.833389135	2.16025E-16	3.79038E-12	1	0.977
fosab	-0.462334437	0.725810867	3.13871E-16	5.50719E-12	0.951	0.841
rps9	-0.529007197	0.693031486	1.04905E-15	1.84066E-11	0.877	0.741
rpl10	-0.576996937	0.670357719	1.46157E-15	2.56447E-11	0.817	0.697
atp5meb	-0.579238562	0.669316942	1.98167E-15	3.47705E-11	0.881	0.725
rps10	-0.524028191	0.695427397	2.13641E-15	3.74855E-11	0.864	0.737
eef1b2	-0.600706877	0.659430775	3.49099E-15	6.12528E-11	0.767	0.657
mybpc3	-0.423013544	0.745865011	3.73221E-15	6.54853E-11	0.928	0.793
rps11	-0.499636238	0.707285094	6.20694E-15	1.08907E-10	0.853	0.728
rpl11	-0.570003841	0.673614995	1.56284E-14	2.74216E-10	0.837	0.712
aldoaa	-0.364184834	0.776907727	1.84135E-14	3.23083E-10	0.976	0.891
faua	-0.572649686	0.672380744	2.33797E-14	4.10221E-10	0.802	0.695
si:ch211-5k11.8	-0.501800783	0.706224716	4.7602E-14	8.35225E-10	0.59	0.339
tpi1b	-0.616094548	0.652434715	7.45752E-14	1.3085E-09	0.81	0.677
rpl8	-0.56437388	0.67624884	1.15146E-13	2.02034E-09	0.842	0.705
hbba1.1	-0.547380139	0.684261585	1.16411E-13	2.04255E-09	0.715	0.415
rpl32	-0.38981465	0.763227654	1.23752E-13	2.17136E-09	0.898	0.792
mt-nd4	-0.361994945	0.778087903	4.58531E-13	8.04539E-09	0.911	0.757
cox7a1	-0.363777746	0.77712698	9.32059E-13	1.63539E-08	0.938	0.832
rps2	-0.31038309	0.806427594	9.6549E-13	1.69405E-08	0.958	0.854
rpl10a	-0.492654315	0.710716297	1.7874E-12	3.13617E-08	0.854	0.716
rps25	-0.43215927	0.741151679	3.46137E-12	6.07331E-08	0.863	0.736
ctrb1	-0.818949979	0.56685436	3.84049E-12	6.73853E-08	0.04	0.119
rpl18	-0.575600339	0.671006972	1.99258E-11	3.49619E-07	0.809	0.674
rps24	-0.351982251	0.783506826	5.36884E-11	9.42018E-07	0.93	0.81
rpl12	-0.471132681	0.721397995	1.18023E-10	2.07082E-06	0.848	0.702
ndufb8	-0.749013923	0.595010106	1.63555E-10	2.86973E-06	0.658	0.573
mt-nd2	-0.329429248	0.795851272	3.17804E-10	5.57619E-06	0.948	0.81
rpl18a	-0.547332934	0.684283974	4.23448E-10	7.42983E-06	0.798	0.664
rbp2a	-0.74376999	0.597176794	4.69574E-10	8.23915E-06	0.048	0.12
acsl1b	-0.982586307	0.506071696	9.48965E-10	1.66505E-05	0.316	0.364

rps8a	-0.470906003	0.721511351	9.80618E-10	1.72059E-05	0.861	0.718
aldoab	-0.612743138	0.653952095	1.31582E-09	2.30874E-05	0.735	0.61
nppa	-0.540792116	0.68739339	1.31644E-09	2.30983E-05	0.856	0.696
socs3a	-0.585899733	0.666233714	2.96872E-09	5.20892E-05	0.787	0.647
RPS17	-0.34652786	0.786474634	3.2276E-09	5.66315E-05	0.906	0.759
rpl15	-0.491182217	0.711441868	6.66172E-09	0.000116887	0.799	0.66
rpl14	-0.351101026	0.783985553	6.91641E-09	0.000121355	0.894	0.743
ndufb10	-0.845554117	0.556497026	5.23817E-08	0.000919089	0.491	0.468
hbba1	-0.557425575	0.679513644	5.28271E-08	0.000926904	0.449	0.283
myl9b	-0.455775778	0.729118	6.25105E-08	0.001096809	0.813	0.647
her4.1	-0.895282256	0.537641998	7.49286E-08	0.001314697	0.649	0.545
hsp90ab1	-0.374315441	0.771471389	1.72425E-07	0.003025373	0.859	0.699
ndufa8	-0.88268434	0.542357359	5.43741E-07	0.009540484	0.526	0.475
rps20	-0.409042883	0.753122847	5.95179E-07	0.010443003	0.8	0.675
pnrc2	-0.613518792	0.653600598	5.95991E-07	0.010457256	0.65	0.553
ndufab1b	-0.861812259	0.550260907	6.14689E-07	0.010785328	0.424	0.422
rbpms2b	-0.693088559	0.61852827	1.14065E-06	0.020013899	0.605	0.518
eef1g	-0.558665368	0.678929949	1.46019E-06	0.025620416	0.736	0.601
atp5mc3a	-0.782190004	0.581483434	2.24978E-06	0.039474563	0.546	0.483

avg_log2FC: average log2 fold change; avg_FC: average fold change; p_val: p-value; p_val_adj: adjusted p-value; pct.1: percentage of the cells in the particular cluster show some expression for this gene; pct.2: percentage of the cells not in the particular cluster (=all the other cells) show some expression for this gene.

SUPPLEMENTARY TABLE S4

Supplemental Table S4. Cluster 2 markers up-regulated (cluster 2 vs cluster 0).

Gene_Symbol	avg_log2FC	avg_FC	p_val	p_val_adj	pct.1	pct.2
zgc:158463	1.991143	3.975519	1.9672E-260	3.4517E-256	0.992	0.748
FO704772.1	1.773238	3.418202	7.3601E-208	1.2914E-203	0.672	0.117
FO704772.2	1.720433	3.295353	3.615E-191	6.3428E-187	0.595	0.094
NC-002333.17	1.494568	2.817797	2.433E-235	4.269E-231	1	0.997
CT027638.1	1.355133	2.558207	2.504E-171	4.3934E-167	0.957	0.645
icn	1.160018	2.234602	1.9951E-115	3.5006E-111	0.64	0.187
serpinh1b	0.942678	1.922093	7.72588E-83	1.35558E-78	0.472	0.131
rn7sk	0.930301	1.905673	6.6412E-109	1.1653E-104	0.65	0.188
CR318588.4	0.846299	1.797882	1.80834E-87	3.17292E-83	0.658	0.237
zgc:86709	0.827908	1.77511	1.36244E-94	2.39053E-90	0.702	0.238
krt18a.1	0.804314	1.746315	3.39376E-49	5.95469E-45	0.271	0.073
NDUFB1	0.786655	1.72507	1.13854E-59	1.99768E-55	0.4	0.128
NC-002333.4	0.76372	1.697863	1.9858E-238	3.4844E-234	1	1
fabp11a	0.719563	1.646683	2.96554E-33	5.20334E-29	0.232	0.075
txn	0.716191	1.642839	2.74646E-57	4.81894E-53	0.371	0.113
s100a10b	0.676244	1.597974	5.28446E-42	9.27211E-38	0.367	0.141
BX548011.2	0.6619	1.582165	1.76592E-46	3.09849E-42	0.234	0.06
postnb	0.65557	1.575239	1.15971E-46	2.03483E-42	0.14	0.014
col1a1b	0.654319	1.573873	2.86317E-46	5.02372E-42	0.212	0.044
tagln	0.649585	1.568716	3.47683E-69	6.10044E-65	0.515	0.174
mdka	0.611517	1.527865	4.9703E-43	8.72089E-39	0.251	0.069
tmsb4x	0.597586	1.513183	1.07009E-53	1.87757E-49	0.934	0.557
sparc	0.572822	1.48743	2.37401E-27	4.16544E-23	0.189	0.061
krt8	0.568232	1.482706	5.56395E-53	9.76251E-49	0.396	0.133
rpl38	0.561207	1.475503	2.57864E-47	4.52448E-43	0.865	0.503
hspb9	0.560672	1.474957	3.15872E-42	5.54229E-38	0.218	0.053
crip1	0.550116	1.464203	3.28047E-27	5.75592E-23	0.184	0.058
rpl26	0.539693	1.453663	1.35978E-55	2.38588E-51	0.579	0.226
mt-atp8	0.535925	1.449871	2.5684E-45	4.50651E-41	0.307	0.1
col1a2	0.532158	1.446091	9.03671E-32	1.58558E-27	0.171	0.042
anxa2a	0.524511	1.438446	1.83739E-63	3.22388E-59	0.636	0.249
si:ch211-222l21.1	0.488444	1.402931	3.50745E-26	6.15417E-22	0.191	0.063
ifitm1	0.47435	1.389292	6.45345E-21	1.13232E-16	0.184	0.07
ppiaa	0.463231	1.378625	1.1365E-35	1.99411E-31	0.35	0.139
acta1a	0.444582	1.36092	7.44647E-51	1.30656E-46	0.641	0.27
col1a1a	0.442904	1.359338	4.51896E-24	7.92896E-20	0.126	0.03
wu:fi09b08	0.439188	1.355841	2.69837E-22	4.73456E-18	0.167	0.06
xirp2a	0.425632	1.34316	6.98541E-26	1.22566E-21	0.224	0.084
hsph1	0.41918	1.337168	3.34965E-42	5.8773E-38	0.766	0.373
cd74a	0.413691	1.33209	7.25024E-31	1.27213E-26	0.183	0.051
krt94	0.405492	1.32454	2.97664E-30	5.22282E-26	0.365	0.16
c6.1	0.405278	1.324344	6.33968E-29	1.11236E-24	0.238	0.086
ccn1	0.405232	1.324301	5.44778E-40	9.55867E-36	0.42	0.167
clu	0.40163	1.320999	1.22757E-36	2.15389E-32	0.328	0.121
cox6a1	0.39697	1.31674	6.31657E-35	1.10831E-30	0.304	0.111
srgn	0.39058	1.31092	6.31674E-18	1.10834E-13	0.141	0.049
uqcrb	0.380605	1.301888	1.08859E-30	1.91004E-26	0.769	0.409
mt-nd3	0.374734	1.2966	1.24247E-33	2.18004E-29	0.808	0.431
sqstm1	0.373615	1.295595	4.44926E-28	7.80667E-24	0.304	0.126

eif5a	0.371629	1.293813	1.0406E-40	1.82583E-36	0.551	0.248
aqp8a.1	0.364937	1.287825	2.91535E-15	5.11528E-11	0.187	0.084
myh7	0.363544	1.286582	4.02179E-43	7.05663E-39	0.804	0.405
fn1b	0.356179	1.280031	3.96623E-17	6.95915E-13	0.164	0.063
ahnak	0.349932	1.2745	1.13933E-21	1.99907E-17	0.189	0.071
clic2	0.339873	1.265646	8.96495E-28	1.57299E-23	0.234	0.084
hsp90b1	0.334203	1.26068	5.35024E-14	9.38754E-10	0.147	0.063
zgc:158343	0.33191	1.258679	5.02107E-18	8.80997E-14	0.152	0.055
atp5mea	0.311625	1.241104	3.38298E-30	5.93578E-26	0.512	0.249
uchl1	0.297187	1.228746	1.67639E-16	2.94139E-12	0.15	0.059
fosl1a	0.292393	1.22467	2.52575E-30	4.43169E-26	0.658	0.333
cd63	0.291528	1.223936	1.19046E-20	2.08878E-16	0.253	0.113
rps27l	0.285187	1.218568	8.79317E-26	1.54285E-21	0.308	0.134
CABZ01102240.1	0.282933	1.216666	5.73074E-25	1.00552E-20	0.356	0.167
rcan1a	0.281078	1.215102	1.35613E-12	2.37946E-08	0.163	0.076
unc45b	0.279189	1.213513	4.26202E-28	7.47813E-24	0.738	0.384
krt15	0.276803	1.211507	6.49213E-20	1.13911E-15	0.222	0.095
rpl6	0.275159	1.210127	7.77523E-23	1.36424E-18	0.851	0.509
fosl2	0.272562	1.207951	9.18317E-14	1.61128E-09	0.136	0.055
sdcbp2	0.271747	1.207269	1.98687E-12	3.48617E-08	0.193	0.099
ctsba	0.270221	1.205993	4.98439E-08	0.000874561	0.118	0.061
dusp5	0.269191	1.205132	2.93366E-14	5.1474E-10	0.152	0.064
slc38a2	0.267506	1.203725	4.13266E-15	7.25117E-11	0.186	0.085

avg_log2FC: average log2 fold change; avg_FC: average fold change; p_val: p-value; p_val_adj: adjusted p-value; pct.1: percentage of the cells in the particular cluster show some expression for this gene; pct.2: percentage of the cells not in the particular cluster (=all the other cells) show some expression for this gene.
